# A novel functionally graded bilayer membrane with excellent barrier function and *in vivo* osteogenesis promotion for guided bone regeneration

**DOI:** 10.3389/fphar.2024.1453036

**Published:** 2024-09-27

**Authors:** Junxuan Li, Jiaxin Ding, Tao Zhou, Bolun Li, Jingjing Wang, Hanchi Wang, Li Fu

**Affiliations:** Department of Oral Implantology, Jilin Provincial Key Laboratory of Tooth Development and Bone Remodeling, Hospital of Stomatology, Jilin University, Changchun, China

**Keywords:** guided bone regeneration, poly(lactic-co-glycolic acid), nanohydroxyapatite, gelatin, osteogenesis

## Abstract

**Introduction:**

Guided bone regeneration (GBR) technology has been widely used as a reliable method to address alveolar bone defects. To improve the clinical effects of GBR approach, there have been attempts to develop barrier membranes with enhanced regenerative properties. However, modifying the material and structure of GBR membranes to integrate physicochemical properties and biological activity remains challenging. The aim of this study was to develop a novel functionally graded bilayer membrane (FGBM) with a gradient structure and composition, and to evaluate its osteogenesis promotion effect for GBR.

**Methods:**

By combining the phase inversion method and electrospinning method, functionally graded bilayer membranes (FGBM) with gradient structure and composition of poly(lactic-co-glycolic acid) (PLGA), nano-hydroxyapatite (nHA), and gelatin were fabricated in this study. The physicochemical and biological properties of the prepared FGBM, including structural and morphological characterization, mechanical properties, *in vitro* biodegradation, cell behaviors, and *in vivo* osteogenic bioactivity, were comprehensively evaluated.

**Results:**

The findings demonstrated the successful fabrication of PLGA/nHA/gelatin FGBM with an asymmetric structure, exhibiting enhanced hydrophilic, mechanical, and degradation properties. The incorporation of gelatin not only improved the biological integration, but also enhanced the binding affinity between electrospun fiber layer and phase inversion layer. The FGBM with a 30% nHA mass fraction and a PLGA/gelatin mass ratio of 1:1 exhibited excellent barrier function and osteogenic bioactivities *in vitro* and *in vivo*.

**Discussion:**

This work demonstrated the potential of PLGA/nHA/gelatin FGBM in bone regeneration and provided valuable insight for the development of barrier membrane.

## 1 Introduction

Insufficient alveolar ridge bone volume poses a significant challenge to the successful placement of implants in the optimal three-dimensional position, leading to adverse effects on the delivery and aesthetic outcome of implant prostheses in the future (Testori et al., 2018; Shi et al., 2022). The guided bone regeneration (GBR) technique is a promising approach to alveolar ridge reconstruction based on the rationale that the regenerative potential of soft tissue exceeds that of bone tissue, and therefore advocates to mechanically prevent the growth of undesirable soft tissues into the bone defect area, thereby allowing only osteoblast clusters from the parent bone to refill (Dahlin et al., 1988; Retzepi and Donos, 2010; Sheikh et al., 2017). In that case, the key element of this technique, guided bone regeneration membrane (GBRM), serves as a biocompatible mechanical barrier that protects the defective area from non-osteoblasts and promotes selective proliferation of autologous osteoblasts, which results in the regeneration of new bone to reconstruct bone defect. The realization of these functions relies on the specific properties of GBRM. Consequently, the choice of GBRM is crucial (Retzepi and Donos, 2010; Elgali et al., 2017; Buser et al., 2023).

According to their degradation performance, GBRM commonly used in clinical practice can be categorized into resorbable and non-resorbable membranes. Non-resorbable membranes are typically composed of polytetrafluoroethylene-based materials or metallic materials with high biocompatibility and mechanical strength (Elgali et al., 2017; Omar et al., 2019; Bee and Hamid, 2022). However, this type of membrane requires a second surgical intervention to be removed, increasing the patients’ therapeutical cost and the risks of wound healing complications (Zhang et al., 2019; Naenni et al., 2021). In comparison, the use of resorbable GBRM eliminates the risk of complications associated with secondary surgery that exists with the non-resorbable GBRM.

The material properties of GBRM are crucial in determining its physicochemical and biological characteristics to meet the requirements of an ideal GBRM (Liu and Li, 2019; Omar et al., 2019; Mizraji et al., 2023). Collagen and synthetic polymers such as polylactic acid (PLA), polyglycolic acid (PGA), poly(lactic-co-glycolic acid) (PLGA), are the most commonly used materials for resorbable GBRM. However, the unpredictable and uncontrolled resorption can lead to the loss of space maintenance of the membranes (Nahid et al., 2022; Patil et al., 2023). In reality, not only is the ideal biodegradation vital for resorbable GBRM, but sufficient mechanical strength may also be beneficial for bone regeneration (Rider et al., 2022). PLGA is an excellent scaffold component for bone tissue engineering materials due to its tunable mechanical strength and processing properties (Lian et al., 2019; Jin et al., 2021). Although its hydrophobic surface structure and lack of cellular recognition sites result in limited cytocompatibility. Additionally, the accumulation of acidic degradation products can cause local inflammation. Fortunately, these issues can be addressed through the implementation of appropriate design and modifications (Jin et al., 2021; Li et al., 2021; Xu et al., 2024). Gelatin is a partially denatured derivative of collagen, inheriting its advantageous properties of good biocompatibility and bioactivity while eliminating its antigenicity. Hydroxyapatite (HA) is the principal inorganic component of bone tissue, exhibiting high biocompatibility and osteoconductive properties (Lin et al., 2020; Zheng et al., 2021; Qin et al., 2023). The incorporation of gelatin and hydroxyapatite into PLGA-based composite scaffolds can compensate for their inherent deficiencies, markedly enhance the mechanical and degradation properties of the composite scaffolds, and augment the osteogenic activity and biocompatibility by mimicking the composition and structure of the ECM, as well as neutralizing the acidic microenvironment generated by PLGA degradation (Ji et al., 2012; Aldana and Abraham, 2017; Naik et al., 2017; Jin et al., 2019). Hence, to maximize the benefits of various biomaterials, researchers have combined gelatin with synthetic polyesters and bioceramic materials to create tissue-engineered nanocomposites with improved physicochemical and biological properties (Fu et al., 2017; Zhang et al., 2020; Gautam et al., 2021; Li et al., 2021; Xiang et al., 2022).

The morphology and structure of GBRM also play a crucial role in its performance and osteogenesis promotion effects. In recent years, a novel functional grade membrane (FGM), characterized by an asymmetric structure and composition, has been developed to further improve the properties (Shah et al., 2019; Abe et al., 2020; Lian et al., 2020). As demonstrated in our previous study (Fu et al., 2017), a functionally graded bilayer membrane (FGBM) with asymmetric bilayer structures, could be fabricated by the phase inversion method combined with electrospinning method, utilizing PLGA and nano-hydroxyapatite (nHA) as primary raw materials. On the one side of the FGBM was a hard and dense layer, which acted as a mechanical barrier to prevent undesirable soft tissues from growing in. On the other side was a porous fiber layer with high porosity and large specific surface area, similar to osteoblastic extracellular matrix (ECM), which could effectively facilitate bone regeneration. However, the hydrophilicity of the PLGA/nHA membrane necessitated improvement. In addition, the biocompatibility and osteogenic activity of the FGBM in animal models remain unexplored.

**FIGURE 1:**
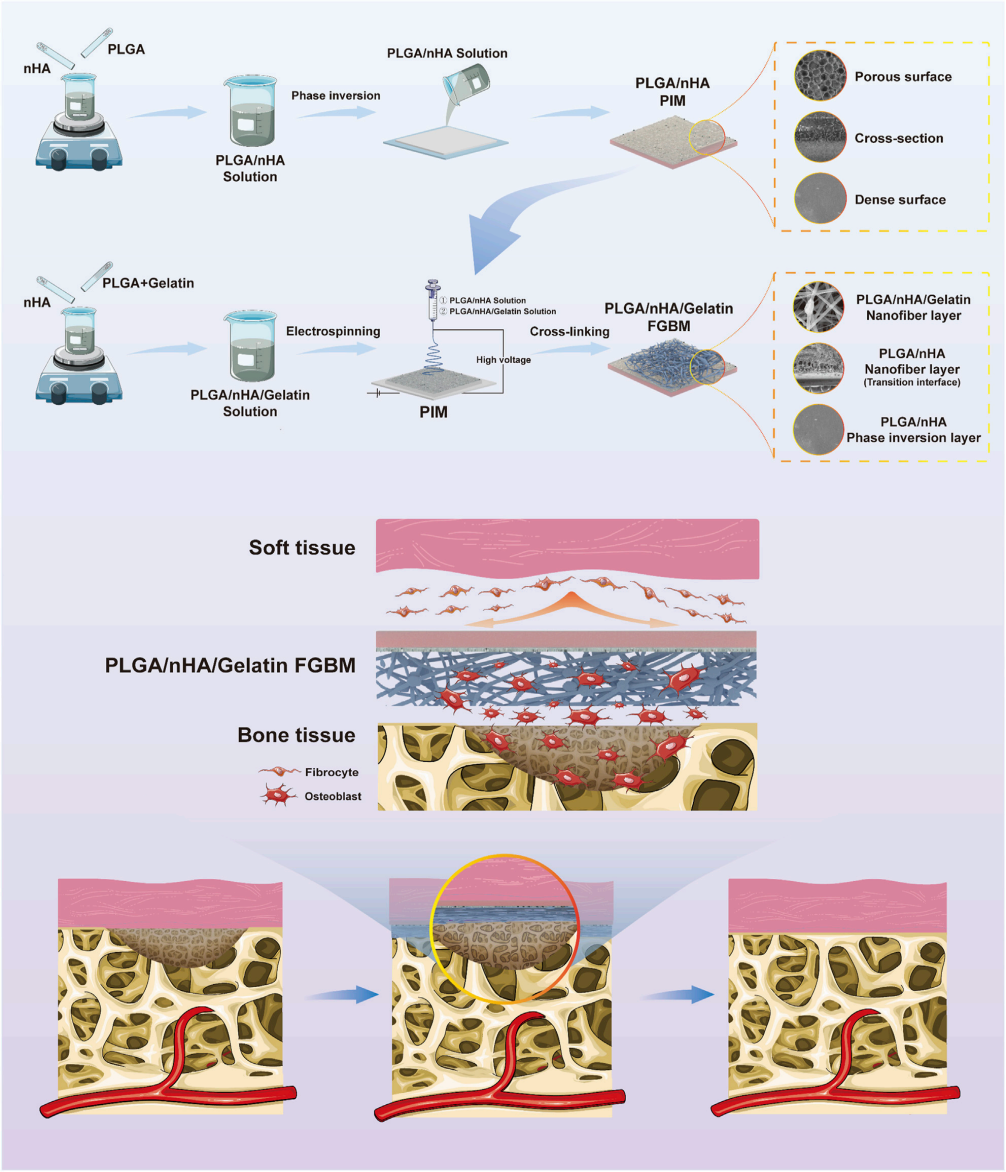
The schematic diagram of the fabrication process of PLGA/nHA/Gelatin functionally graded bilayer membrane and applied for guided bone regeneration.

To address these gaps, further improve the physicochemical properties of FGBM and explore their osteogenesis promotion effects *in vivo*, the present study drew inspiration from bionics and further modified FGBM by mimicking the composition and structure of the osteoblastic ECM (Figure 1). As widely acknowledged, in the osteoblastic ECM of natural bone tissue, the deposition of calcium is deposited on collagen fibers as the form of hydroxyapatite (HA), which together provide structural support for bone tissue (Salhotra et al., 2020). In this study, using bioactive materials that are fully biocompatible as raw materials, we introduced gelatin into the primary PLGA/nHA nanofibers, which were then electrospun onto the bone-tissue interface of a PLGA/nHA phase inversion membrane, to fabricate a PLGA/nHA/Gelatin FGBM. nHA particles were uniformly doped into the individual nanofiber scaffolds composed of gelatin and PLGA as the main body. Such nanofibers were interwoven with each other to fully utilize the advantages of this strategic modification in terms of physical structure and chemical composition. This enhanced the mechanical strength of the whole membrane while facilitating osteoblast adhesion and growth by providing suitable carriers and stimulation signals, thereby promoting cell proliferation and differentiation. A series of *in vitro* and *in vivo* experiments were designed and performed to validate the dual effects of the good tissue barrier and bone-enhancing effects of PLGA/nHA/Gelatin FGBM. These results are encouraging, as they indicate a promising future for PLGA/nHA/Gelatin FGBM in the treatment of alveolar bone defects.

## 2 Materials and methods

### 2.1 Materials

Nano-Hydroxyapatite (nHA, 50–100 nm in length and 20–30 nm in width), N, N-dimethylformamide (DMF), and 1,1,1,3,3,3-Hexafluoro-2-propanol (HFIP), were purchased from Aladdin (China). Gelatin, 3-(4,5 dimethylthiazol-2-yl)-2,5-diphenyl tetrazolium bromide (MTT), 4′,6-diamidino-2-phenylindole (DAPI), trypan blue solution, and cetylpyridinium chloride were purchased from Sigma-Aldrich (United States). Poly(Lactic-co-glycolic acid) (PLGA, LA: GA = 75:25, with a molecular weight of 100,000) was donated by the Changchun Institute of applied chemistry (Changchun, China). Dulbecco’s Modified Eagle Medium (DMEM), 10% fetal bovine serum (FBS), and Penicillin streptomycin were purchased from Gibco (United States). Trypsin was purchased from Biotechs (China).

### 2.2 Fabrication of PLGA/nHA/gelatin functionally graded bilayer membrane

#### 2.2.1 Fabrication of phase inversion membrane

The fabrication process of the phase inversion membrane closely followed the procedures outlined in our prior study (Fu et al., 2017). PLGA was dissolved in N,N-dimethylformamide (DMF) to prepare a 5% (w/v) homogeneous solution. nHA was added to the mixture in an nHA/(PLGA + nHA) mass fractions of 5%. The solution was then stirred vigorously for 2 h to ensure homogeneous dispersion of the nHA. Subsequently, the solution was casted onto a glass plate maintained at a constant temperature and scraped with a spatula. The glass plate was then submerged in a water bath at 25°C until the film was fully detached to obtain a phase inversion membrane (PLGA with 5 wt% nHA) (PIM).

#### 2.2.2 Preparation of PLGA/nHA electrospinning solution

1 g PLGA was added in 6 mL HFIP and stirred magnetically until complete dissolution. Add 0.43 g nano-hydroxyapatite (nHA/(PLGA + nHA) = 30 wt%) into 4 mL HFIP and ultrasonically dispersed for 30 min to disperse nHA particles uniformly. Blend the above two solutions and stir continuously for 24 h to get PLGA/nHA electrospinning solution.

#### 2.2.3 Preparation of PLGA/nHA/gelatin electrospinning solution

PLGA and Gelatin were dissolved in 2 mL HFIP and stirred until completely dissolved, respectively. Blended the two solutions, then added 12 μL acetic acid (CH_3_COOH), and stirring was continued for 6 h. The ratios of PLGA/Gelatin (w/w) were set to 7/3 (0.42 g PLGA, 0.18 g Gelatin), 5/5 (0.3 g PLGA, 0.3 g Gelatin), and 3/7 (0.18 g PLGA, 0.42 g Gelatin). 0.25 g of nano-hydroxyapatite [nHA/(PLGA + Gelatin + nHA) = 30 wt%] was added into 2 mL HFIP and ultrasonically dispersed for 30 min. The above two solutions were blended and stirred for 24 h to obtain three types of PLGA/nHA/Gelatin electrospinning solutions.

#### 2.2.4 Electrospinning

The phase inversion membrane was fixed on a 10 cm × 10 cm aluminum foil receiving screen with the rough surface facing upward. Electrospinning was performed at room temperature with a voltage of 20 kV, a spinneret diameter of 0.4 mm, a flow rate of 1 mL/h, and a receiving distance of 15 cm, utilizing a high-voltage Electrospinning machine. Initially, 1 mL of PLGA/nHA solution was electrospun onto the phase inversion membrane, followed by the immediate electrospinning of 6 mL of PLGA/nHA/Gelatin solution onto the PLGA/nHA layer. The electrospinning process was repeated three times to produce three distinct types of functionally graded bilayer membrane, FGBM PHG1, FGBM PHG2, and FGBM PHG3, each composed of different ratios (7/3, 5/5, and 3/7) of PLGA/Gelatin. These membranes underwent cross-linking in glutaraldehyde (GA) vapor for 1 h. In addition, the remaining 7 mL of PLGA/nHA solution was electrospun onto the phase inversion membrane alone, serving as a control group (FGBM PH). Subsequently, the four obtained FGBMs were dried in a vacuum oven (Jinghong, Shanghai, China) for 24 h to eliminate residual solvent.

### 2.3 Structural and morphological characterization methods

Structural and morphological characterizations of PLGA/nHA/Gelatin electrospun fiber layer of FGBMs (FGBM PH, FGBM PHG1, FGBM PHG2, and FGBM PHG3) and PIM were observed by environmental scanning electron microscope (ESEM; Model XL 30 ESEM FEG, Micro FEI Philips, Amsterdam, Netherlands) and transmission electron microscopy (TEM, JEM 200cx, Japan). The hydrophilicity of the electrospun fiber layer of FGBM was evaluated by a contact angle tester (Fangrui, Shanghai, China) at room temperature.

### 2.4 Mechanical tests

The mechanical properties of FGBM were evaluated by Instron 3,367 mechanical testing machine (Norwood, United States). Each type of membrane was cut into 20 mm × 15 mm rectangular samples. Measured the sample thickness with a spiral micrometer. Stretched the sample at a tensile strength of 100 N and a speed of 10 mm ·min^−1^, at room temperature. The values of tensile strength were recorded.

### 2.5 *In vitro* biodegradation

To evaluate the *in vitro* biodegradation performance of FGBM, rectangular samples of 8.0 mm × 5.0 mm were prepared. Each sample’s initial weight ( $W_{0}$ ) was measured separately on an analytical balance. The samples were immersed in 10 mL phosphate-buffered saline (PBS) (0.1 M, pH = 7.4), and placed in a constant temperature oscillating incubator (100 rpm) at 37°C. Weekly, removed the samples, quickly absorbed surface water with filter paper, and then weighed on an analytical balance ( $W_{t}$ ). Then placed the samples in a desiccator to dry completely, and weighed on an analytical balance ( $W_{t}^{\prime }$ ). The mass remaining (percentage) was calculated by Equation 1, and the water uptake (percentage) was calculated by Equation 2. Measured the mass retention, water uptake, and pH of the samples weekly for 8 weeks.

$$\text{Mass remaining }\left(\%\right)=W_{t}^{\prime }/W_{0}\times 100\;\%$$ (1)

$$\text{Water uptake }\left(\%\right)=\left(W_{t}-W_{t}^{\prime }\right)/\;W_{t}\times 100\;\%$$ (2)

### 2.6 *In vitro* biocompatibility

#### 2.6.1 Barrier function of phase inversion membrane to L929 cells

The barrier function of PIM was *in vitro* evaluated on L929 cells according to previously established method (Fu et al., 2017). To briefly summarize, the PIM was immobilized using a Cell Crown (Sigma-Aldrich, St. Louis, MO, United States), and a L929 cells suspension at a density of 4×10^3^ cells·ml^-1^ was added to the Cell Crown and suspended in a 24-well plate spiked with 1 mL of cell-free medium liquid. The smooth surface of the membrane was kept in direct contact with the cells during the process (Supplementary Figure S4A). At 1 and 3 days after incubation, the smooth surface of the membrane and the bottom of the 24-well plate were stained with DAPI. Cell growth on the membrane and at the bottom of the well plate was observed using an inverted fluorescence microscope (TE2000-S, Nikon, United States).

#### 2.6.2 MC3T3-E1 cells adhesion on the FGBM

The FGBM was cut to the size of the well of 24-well cell culture plate, and the UV light irradiated the front and back of the FGBM samples for 1 h, respectively. The sterilized sample was then placed inside the 24-well plate with the porous fiber layer facing upward, and a sterile iron ring with an inner diameter matching the diameter of the 24-well plate was pressed onto the sample to prevent it from floating up. MC3T3-E1 cells were inoculated on the fib surface at a density of 1.5×10^5^ cell·cm^-2^ and cultured at 37°C with 5% CO_2_, using CO_2_ thermostat cell incubator (MCO-15AC, SANYO, Japan). To detect the early adhesion of cells, at 4 h after inoculation, stained the cells with DAPI, and then observed the cells cultured on the scaffold under inverted fluorescence microscope. The cell density (D) was calculated by D_cell_ = N_cell_/A (N_cell_ represented the number of cells in the observation area, and A represented the area of the observation area). Six parallel samples were tested in each group. Three observation areas were randomly selected for each sample for photograph and calculation.

#### 2.6.3 MC3T3-E1 cells proliferation on the FGBM

Cell behaviors were evaluated by detecting the survival of Murine osteoblast-like cells (MC3T3-E1 cells) inoculated on FGBM. The cells were donated by the Medical Department of Jilin University (Changchun, China). MC3T3-E1 cells were inoculated on the fibrous surface of FGBM at a density of 1.5×10^5^ cells/cm^2^. The culture was terminated at 1, 4, and 7 days, separately, and the cells that failed to attach to the membrane were washed three times with PBS.

The integrity of the cytomembrane was examined using a live/dead cell staining assay to evaluate cell viability on the nanofiber membrane. Covered the surface of the samples with PBS which contained 4 μM ethidium homodimer^−1^ (EthD^−1^) and 2 μM calcein-AM, and stained the cells for 20 min. The stained cells were observed under an inverted fluorescence microscope, in which live cells were excited by blue light fluorescing green and dead cells excited by green light fluorescing red. The percentage of live cells was calculated at each time point.

The MTT was used to detect cell proliferation on the nanofiber membrane. Added 20 μL of 5 mg mL^-1^ MTT solution to the well and incubated at 37°C for 4 h. Then carefully removed the supernatant, added 150 μL of LDMSO, shook it to dissolve the formed methanogenic crystals completely. The levels of MTT were determined by measuring the absorbance value at 490 nm with a Microplate Reader (SANYO, Japan).

#### 2.6.4 MC3T3-E1 cells differentiation on the FGBM

The differentiation of MC3T3-E1 cells on the PLGA/nHA/Gelatin electrospun fiber layer was assessed through an alkaline phosphatase (ALP) activity assay. MC3T3-E1 cells were seeded onto the fibrous surface of the FGBM at a density of 1.5×10^5^ cells/cm^2^. The culture was terminated at two distinct time points, namely, 1 and 14 days. At each time point, cells were lysed, and the collected lysate was subjected to relative quantitative measurements of ALP using an ALP substrate reaction solution. The levels of ALP were determined by measuring the absorbance value at 405 nm with a Microplate Reader.

Alizarin red S (ARS) staining was used to assess the quality and quantity of the extracellular matrix secreted by MC3T3-E1 cells. At 1, 7, and 14 days of cell culture, the cell-laden scaffolds were fixed in 90% ice ethanol solution for 10 min, then washed 3 times with distilled water, to which the configured 0.1% ARS dye was added, stained in a 37°C water bath for 30 min, gently washed three times with deionized water, and photographed by a camera. Then the ARS on the specimen was dissolved with 10% cetylpyridinium chloride. The levels of ARS were determined by measuring the absorbance value at 540 nm with a Microplate Reader.

### 2.7 *In vivo* assessments with rat cranial bone defect model

#### 2.7.1 Rat cranial bone defect model establishment

Twenty-four Wistar rats, aged 12 weeks and weighing between 250 and 300 g, were selected for the establishment of 5-mm cranial bone defects, assessing the bone-regenerating potential of the PLGA/nHA/Gelatin FGBM. Ethical guidelines were strictly observed in accordance with the approval of the Animal Experimentation Ethics Committee of the Stomatology School of Jilin University.

Cranial bone defects were induced using a trephine bar (Micro-Tech, Japan) in accordance with a previously described method (Yoshimoto et al., 2018). The rats were randomly divided into four groups based on the type of membrane used. The test group, which evaluated *in vivo* bone regeneration, featured the PLGA/nHA/Gelatin FGBM with PLGA/Gelatin ratios of 1:1 (FGBM PHG2). The control groups included a blank group, a PLGA/nHA FGBM group (FGBM PH), and a Bio-Gide^®^ group. The membranes (FGBM PH, FGBM PHG2, and Bio-Gide^®^) were cut into 5.0 mm diameter circular pieces and applied to cover the bone defects. The blank controls had no membrane coverage. Following the application of sutures to the periosteum and scalp, the rats resumed normal activity following the recovery period. At 4 and 8 weeks, the rats were euthanized, and the cranial areas were harvested and fixed with 10% formalin for subsequent analysis.

#### 2.7.2 Micro-computed tomography (Micro-CT) measurement

Micro-CT (SCAN CO, Switzerland) was used to monitor the healing of the bone defect area and to quantitatively assess the volume of regenerated bone. A simultaneous 3D reconstruction was performed through scanning with standardized segmentation parameters (sigma: 0.8, threshold value: 220–1,000). Circular contour lines, excluding the adjacent native bone, were delineated around the 5 mm diameter defect area. The machine’s built-in software generated 3D reconstructed images from 2D slices. Quantitative outcomes were expressed as a percentage of bone volume to tissue volume.

#### 2.7.3 Histological and histomorphometric observation

Specimens underwent decalcification using 20% EDTA for a period of 3 months. Following gradient dehydration in ethanol, the samples were embedded in paraffin and sectioned in a coronal plane, producing slices with a thickness of 5 μm. The specimens were subjected to Hematoxylin-Eosin staining (H&E) and microscopically observed to assess the regenerated bone.

### 2.8 Statistical analysis

All experiments were repeated three times, and the data obtained were expressed as the mean ± standard deviation (SD). Overall, the statistically significant values were estimated using two-sided Student’s t-tests and one-way analysis of variance (ANOVA) tests. Statistical analyses were performed using SPSS 19.0 software (SPSS, Chicago, IL, United States). A P-value less than 0.05 was considered statistical significance. In the figures, letters were used to show statistical differences, and values with dissimilar letters are significantly different from each other (p < 0.05).

## 3 Results

### 3.1 Morphology observation of FGBMs

**FIGURE 2:**
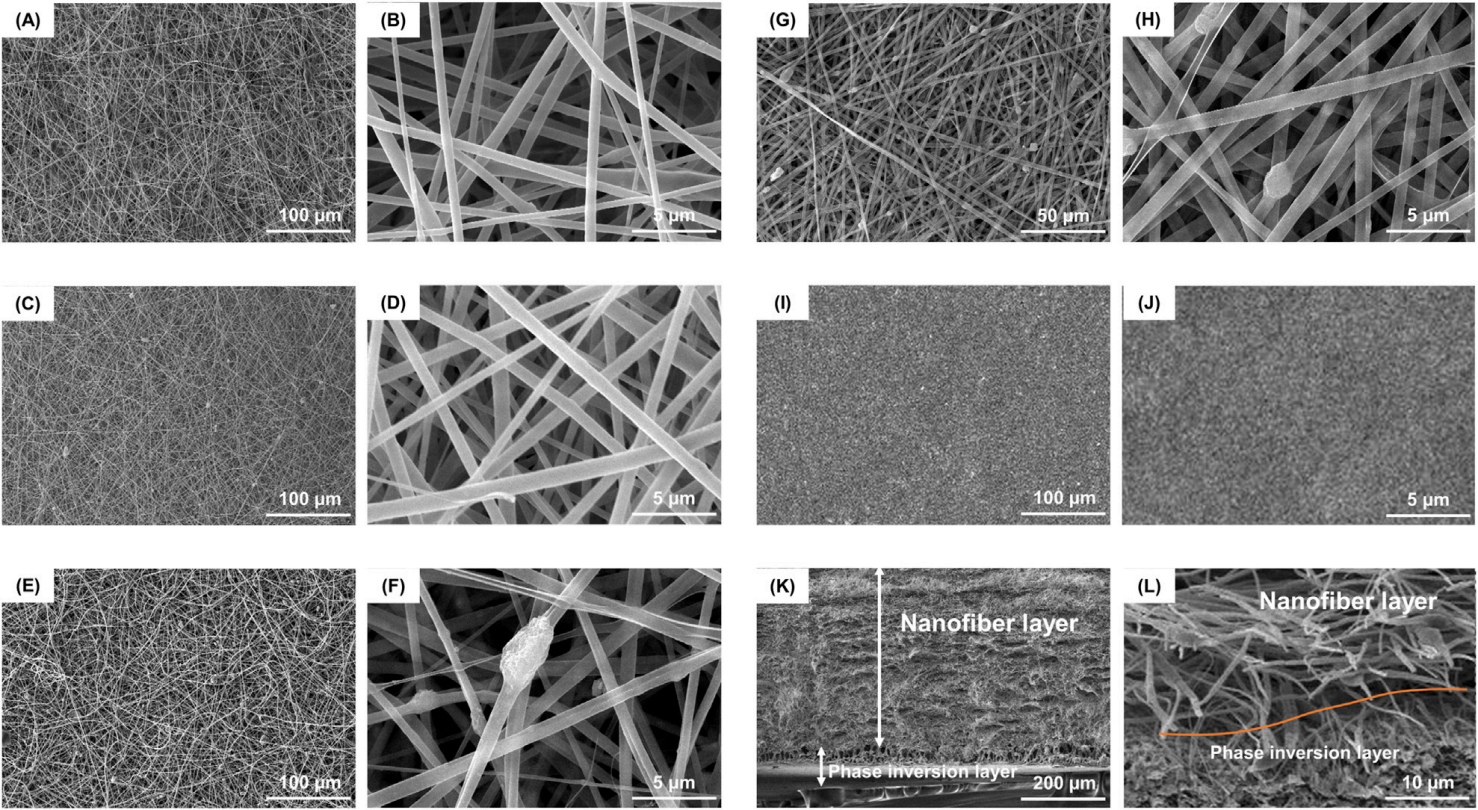
Representative SEM images of surface morphology and structure of different FGBMs. **(A, B)** FGBM PH. **(C, D)** FGBM PHG1. **(E, F)** FGBM PHG2. **(G, H)** FGBM PHG3. **(I, J)** The dense surface of PIM. **(K)** The cross-sectional thickness of the FGBM was about 600 μm, in which the phase inversion layer was about 100 μm and the nanofiber layer was about 500 μm. **(L)** The red curve indicated the boundary between the phase inversion layer and the nanofiber layer. The interlayer was tightly bonded.

PLGA/nHA/Gelatin FGBM was successfully prepared (Supplementary Figure S1). The surface morphology of FGBM was examined using environmental scanning electron microscopy (ESEM) and transmission electron microscopy (TEM). The SEM images indicated the porous interconnected fibrous structures of nanofiber layers (Figures 2A–H). The TEM observations showed that the PLGA/nHA/Gelatin fibers had a smooth and continuous appearance, as well as a uniform distribution of nHA particles without any discernible defects (Supplementary Figure S2A, B). The energy-dispersive X-ray (EDX) spectrum and elemental mapping image of PLGA/nHA/Gelatin nanofiber layer displayed the proportion and distribution of phosphorus (P) and calcium (Ca), indicating the uniform dispersion of nHA on the FGBM (Supplementary Figure S2C). The fiber diameters for FGBM PH, FGBM PHG1, FGBM PHG2, and FGBM PHG3 were 1,145 ± 450 nm, 763 ± 267 nm, 653 ± 416 nm, and 922 ± 584 nm, respectively. Post gelatin incorporation, fiber diameters decreased compared to FGBM PH, subsequently increasing with higher gelatin proportions. nHA was evenly distributed on the fiber surface, forming protuberant structures locally due to nanoparticle aggregation, which increased with higher gelatin proportions. The individual phase inversion membrane (PIM) exhibited an asymmetric structure comprising a dense outer smooth surface and a porous inner rough surface (Figures 2I, J, S3).

Figures 2K, L illustrates the cross-sectional morphology of FGBM PHG3, resembling that of other FGBM. The cross-sectional thickness of FGBM measured approximately 600 μm, comprising a 100 μm phase inversion layer and a 500 μm nanofiber layer. In Figure 2K, both the dense layer and porous layer belonging to the phase inversion layer. The red curve depicted in Figure 2L indicates the interface between the phase inversion layer and the nanofiber layer, tightly bonded. The PLGA/nHA transition fiber layer bridged the phase inversion layer and electrospun fiber layer without obvious boundaries.

### 3.2 Contact angle analysis of FGBMs

**FIGURE 3:**
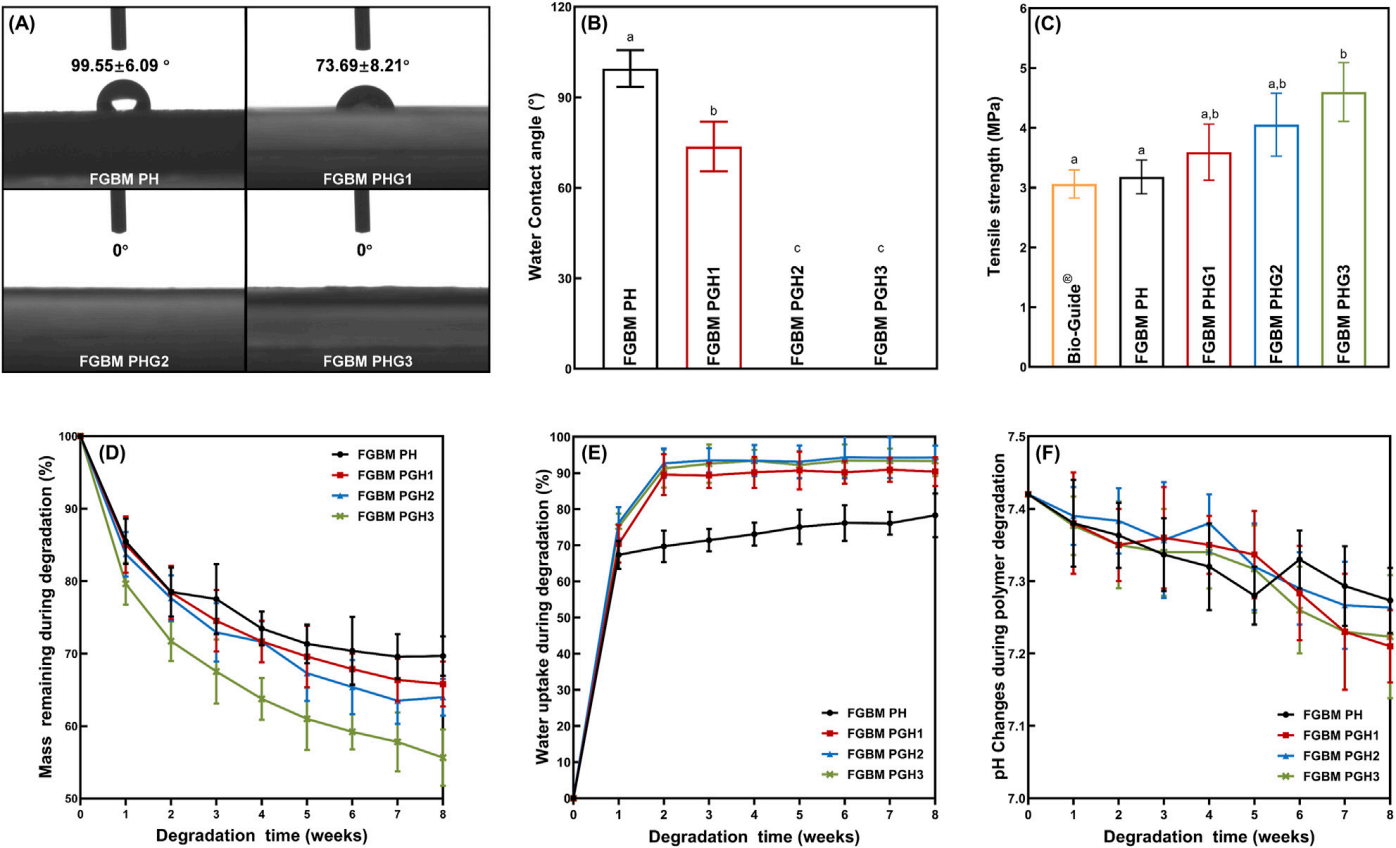
*In vitro* evaluation of physicochemical properties. **(A, B)** The water contact angle of the fiber surface of four groups of the FGBMs. **(C)** Means (Standard deviations) of tensile strength of Bio-Gide^®^ (commercial control) and four groups of FGBM with different compositions. **(D)** Mass remaining during degradation (%) of the FGBMs with different compositions. **(E)** Water uptake during degradation (%) of the FGBMs with different compositions. **(F)** pH changes during polymer degradation of the FGBMs with different compositions. Different letters above the bar graph represent statistically significant differences among groups (*P* < 0.05).

The contact angle is a common index used to measure the hydrophilicity of materials. As depicted in Figures 3A,B, after incorporating gelatin into the fiber layer, water droplets spread out immediately upon contact with the fiber surface, and the water contact angle significantly reduced compared to that of the fiber without gelatin, reaching 0° (*P*< 0.05). With the increase in gelatin content, the water contact angle decreased noticeably. In the FGBM PHG2 and FGBM PHG3 groups, water droplets disappeared completely upon contact with the membranes, resulting in contact angles of 0° for both groups. These results were notably different from the FGBM PHG1 group.

### 3.3 Mechanical properties of FGBMs

FIGURE 3C illustrates the tensile strength of four types of PLGA/nHA/Gelatin FGBM and commercial collagen membranes. The tensile strength of the FGBM increased with the rise in gelatin content. FGBM PHG3 exhibited the highest tensile strength, measuring 4.60 Mpa, with statistically significant differences compared to FGBM PH (3.18 Mpa) and Bio-Gide^®^ (3.06 Mpa) (*P*< 0.05). Conversely, no statistically significant differences were observed between FGBM PHG1 (3.59 Mpa) and FGBM PHG2 (4.05 Mpa) (*P*> 0.05).

### 3.4 *In vitro* biodegradation of PLGA/nHA/gelatin FGBM

FIGURE 3D-F depicts the *in vitro* biodegradation results of four types of FGBM during the degradation period. The mass remaining increased at each time point, with FGBM PHG3 degrading the fastest (Figure 3D). By the 8th week, the mass of FGBM PHG3 had decreased by nearly half of its initial weight, yet the membrane morphology remained intact due to the support of the phase inversion layer. The performance and rate of water uptake of the gelatin-containing membranes increased significantly (Figure 3E). Within the first 2 weeks, the water uptake percentage of the membranes rapidly reached approximately 90%, followed by a gradual increase over the subsequent period. In contrast, the water uptake percentage of FGBM PH, the membrane without gelatin, was close to 70% at 2 weeks and increased slowly thereafter. The pH values of all four types of FGBM experienced a slight decrease at 8 weeks, yet they remained within the neutral range (Figure 3F).

### 3.5 *In vitro* barrier function of phase inversion membrane to L929 cells

The barrier effect of the PIM on L929 cells was evaluated *in vitro*. Supplementary Figure S4 illustrates the infiltration of L929 cells cultured on the smooth side (inside) of the phase inversion membrane to the other side of the membrane (outside). During the 1-day and 3-day incubation periods, the number of cells on the smooth side of the membrane increased, while only a minimal number of cells migrated into the medium under the membrane. This indicated that the PIM exhibited a superior barrier effect on L929 cells.

### 3.6 *In vitro* cell adhesion on the FGBM

The fluorescence micrographs of the early adhesion of MC3T3-E1 cells on the electrospun fiber layer of FGBM after cultured for 4 h are illustrated in Supplementary Figure S5A–D. Quantitative analysis of cell density (Supplementary Figure S5E) showed that the cells adhered on the surface of FGBM PHG2 and FGBM PHG3 were significantly more than the other two groups (*P*< 0.05), indicating that gelatin promoted the adhesion of osteoblasts on the membrane surface.

### 3.7 *In vitro* cell proliferation on the FGBM

**FIGURE 4:**
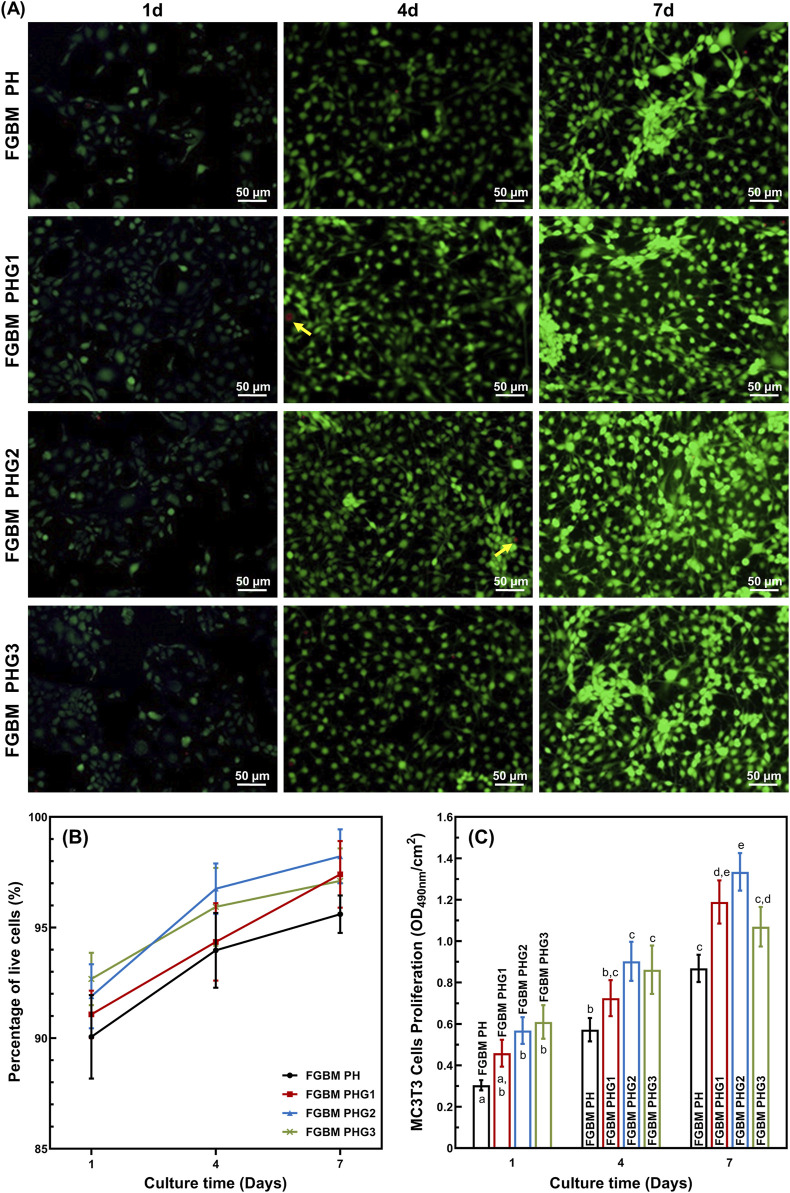
*In vitro* cellular proliferation activity of MC3T3-E1 cells on the electrospun fiber layers of the FGBMs. **(A)** Live/dead staining results of MC3T3-E1 cells of the four groups of FGBM at 1, 4 and 7 days. Green fluorescence is indicative of live cells, whereas red fluorescence (yellow arrows) is indicative of dead cells. **(B)** Percentage of live cells on the FGBM of the four groups. **(C)** Proliferative activity of MC3T3-E1 cells on FGBM of the four groups Different letters above the bar graph represent statistically significant differences among groups (*P* < 0.05).

The cell proliferation behavior of MC3T3-E1 cells on the electrospun fiber layer of FGBM are illustrated in Figure 4. Fluorescence micrographs of live and dead cell staining are presented in Figure 4A. Notably, the number of live cells (green fluorescence) increased significantly with prolonged culture time, with scattered dead cells (red fluorescence) observed. The percentages of live cells in all groups were consistently high, particularly in the FGBM groups containing gelatin. Cell viability increased with culture time. From 1 to 7 days during the culture period, the percentage of live cells in each group was higher than 90% with no statistical difference among the groups. Specifically, at 7 days, the number of live cells on FGBM PHG2 and FGBM PHG3 was significantly higher than that on FGBM PH and FGBM PHG1 (Figure 4B).

MTT assay of MC3T3-E1 cells was employed to evaluate cell viability. The results of the MTT assay indicated that cell viability of each group increased significantly during the culture period from the 1st to the 7th day (Figure 4C). At all time points during the culture period, the control group without gelatin incorporation was significantly lower than those of the other groups (*P*< 0.05). These findings suggest that PLGA/nHA/Gelatin composite fibers exhibit good cytocompatibility, and the introduction of gelatin can promote the proliferation of MC3T3-E1 cells.

### 3.8 *In vitro* cell differentiation on the FGBM

**FIGURE 5:**
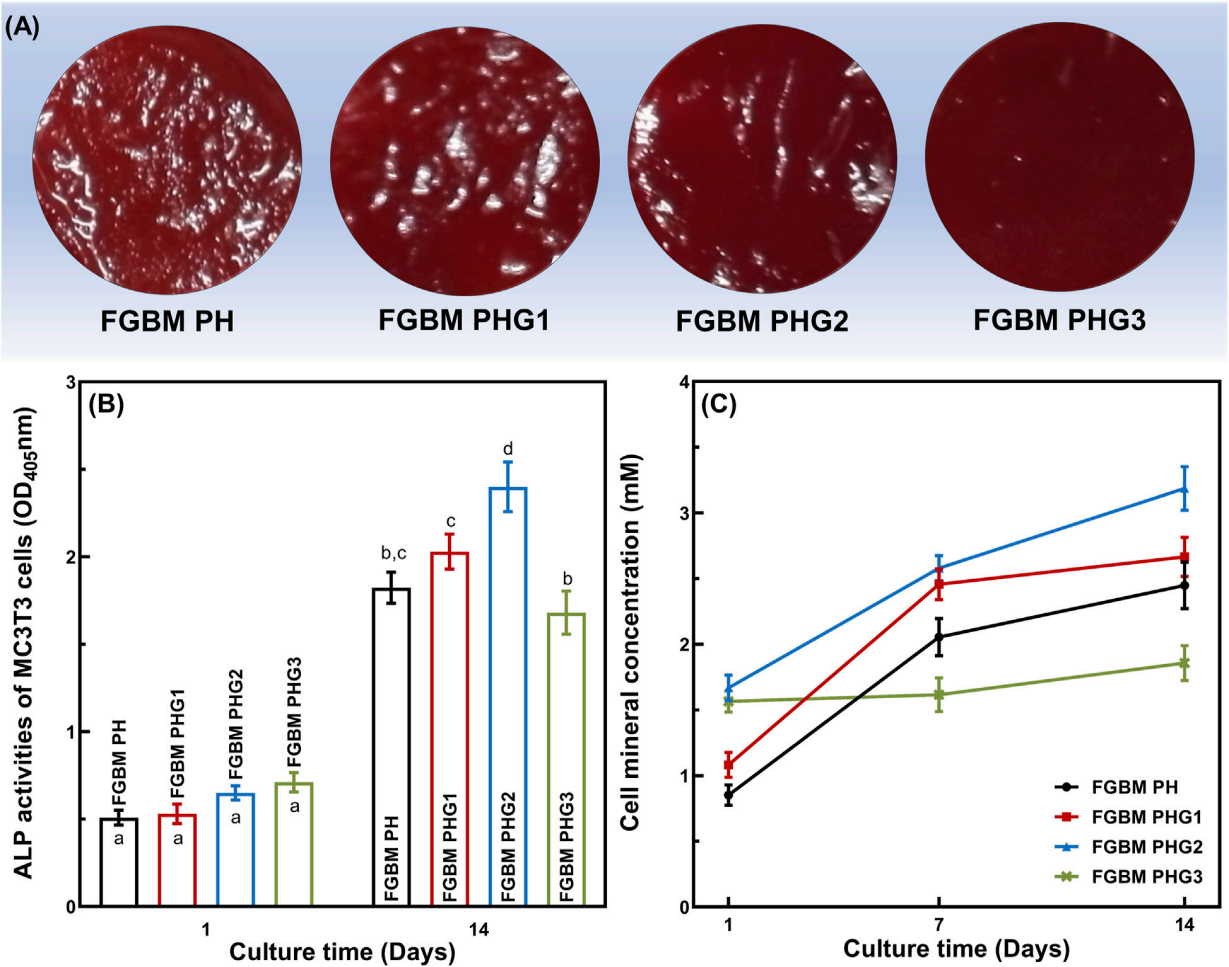
*In vitro* cellular differentiation activity of MC3T3-E1 cells on the electrospun fiber layer of the FGBMs. **(A)** The alizarin red S staining results of MC3T3-E1 cells at 14 days, respectively. **(B)** ALP activity of MC3T3-E1 cells on the FGBM at 1 and 14 days. **(C)** Quantitative analysis of cell mineral concentration of MC3T3-E1 cells on the FGBM of the four groups at 1, 7, and 14 days. Different letters above the bar graph represent statistically significant differences among groups (*P* < 0.05).

The effects of four types of FGBM on the differentiation of MC3T3-E1 cells are depicted in Figure 5. ALP is an early marker of osteoblast differentiation. Figure 5B shows the ALP activity measured after 1 and 14 days of MC3T3-E1 cell culture on PLGA/nHA/Gelatin FGBM. As observed, the ALP activity was at a similar level among the groups on the 1st day. After being cultured for 14 days, the ALP activity increased significantly (*P*< 0.05) in each group compared to the 1st day, indicating the excellent osteogenic effect of FGBM. During the culture period, with the increase of gelatin content, the ALP secretion increased at first and then decreased. Additionally, the ALP activity of the FGBM PHG2 group was significantly higher than that of the other three groups (*P*< 0.05).

For cytosolic mineral deposition of cells, ARS and the corresponding semi-quantitative method were used to assess the ability of four types of FGBM to induce osteogenic differentiation and mineral deposition. As shown in Figure 5A, there was an apparent deposition of calcium salt on all PLGA/nHA/Gelatin fibers at 14 days. Furthermore, the evaluation of calcium salt deposition confirmed the microscopic ARS staining results, with the FGBM PHG2 group exhibiting the highest cell mineral concentration, whereas the FGBM PHG3 group showed the lowest (Figure 5C).

### 3.9 Micro-computed tomography (Micro-CT) measurement

**FIGURE 6:**
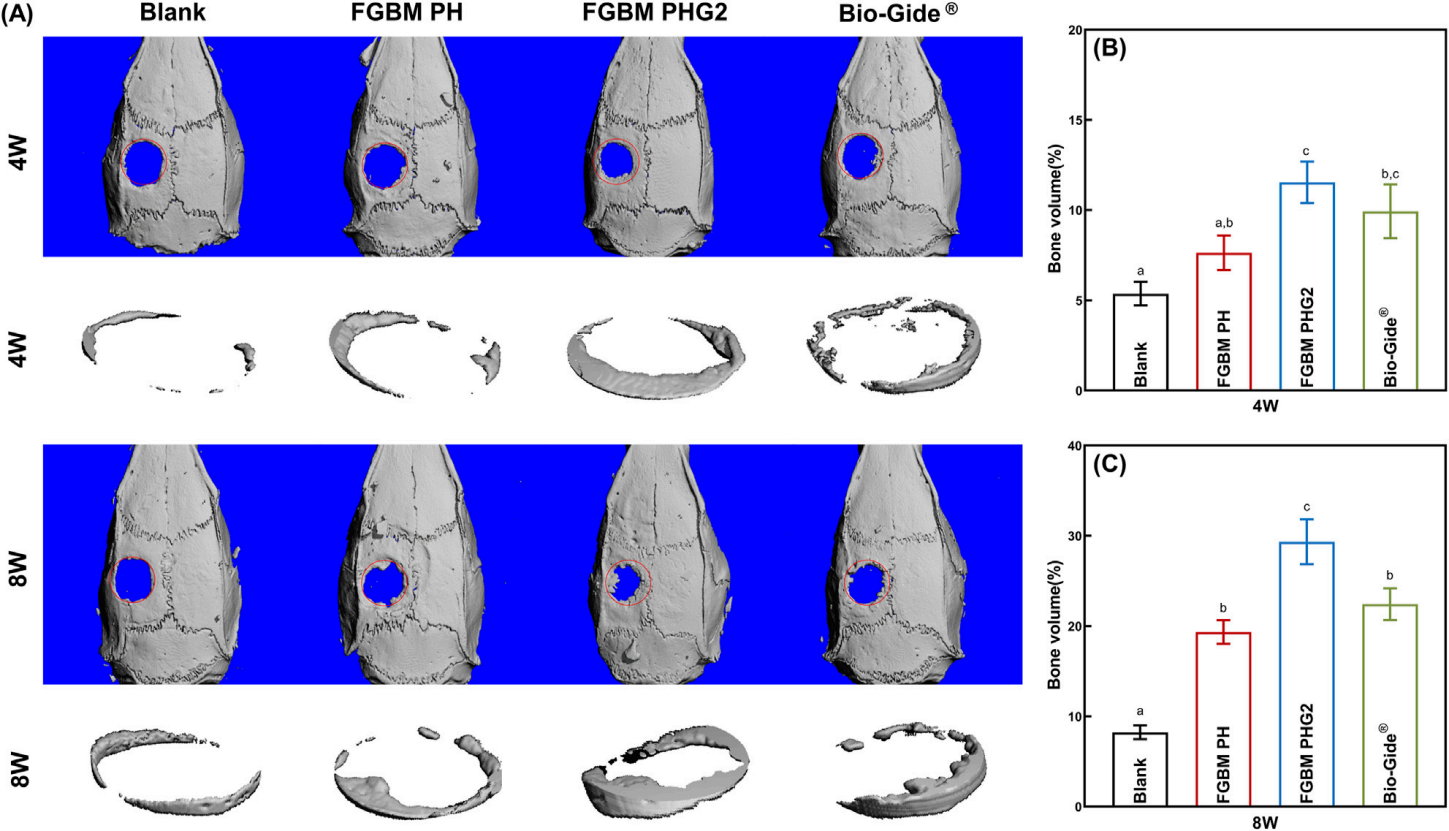
Micro-CT analysis of the bone defect areas after 4 and 8 weeks of implantation of FGBMs and commercial collagen membrane in rat cranial defect models. **(A)** Three-dimensional reconstructed images of micro-CT of the cranial defect area of rat models: new bone formation in the bone defect after 4–8-week implantation of the membranes. **(B)** The volume of bone regeneration in the defect area the of the rat models after 4-week implantation of the membranes. **(C)** The volume of bone regeneration in the defect area of the rat models after 8-week implantation of the membranes. Different letters above the bar graph represent statistically significant differences among groups (*P* < 0.05).

Micro-CT was employed to assess the impact of the prepared FGBM on bone defect regeneration. As shown in Figure 6A, at 4 and 8 weeks, the FGBM PHG2 group exhibited the greatest regenerated bone area. At 4 weeks, the FGBM PHG2 group exhibited a regenerated new bone volume of approximately 12%, which was significantly greater than that observed in the blank control and FGBM PH groups (Figure 6B). At 8 weeks, only a trace bone formation was observed at the edge of the defect area in the blank control group. When compared to the blank control group, the extent of the defect areas was significantly reduced in the three membrane-covered groups. Among the groups, the FGBM PHG2 group exhibited the highest bone volume of regenerated new bone, reaching nearly 30%. This value was significantly higher than that observed in the other groups (*P*< 0.05) (Figure 6C).

### 3.10 Histological observation

**FIGURE 7:**
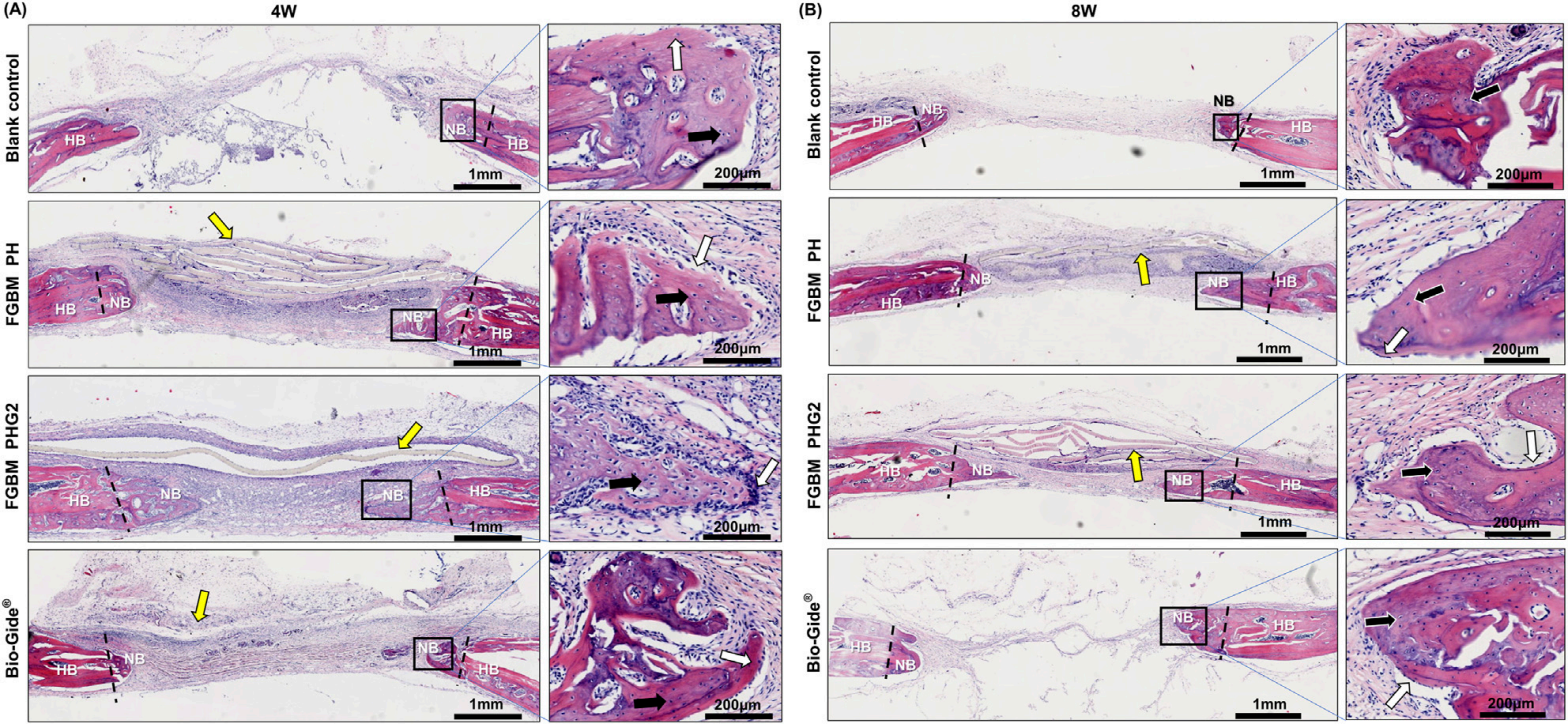
Representative H&E staining images of the bone defect areas after 4 and 8 weeks of implantation of FGBMs and commercial collagen membrane in rat cranial defect models. **(A, B)** H&E staining images of regenerated bone tissue after **(A)** 4-week and **(B)** 8-week implantation of the membranes. The periosteum side is on top, and the dura mater side is on the bottom (left part of **(A, B)**, scale bar = 1 mm), respectively. The black rectangle areas were magnified on the right (right part of **(A, B)**, scale bar = 200 μm), respectively. The boundary between the new bone and the host bone is marked by black dotted lines, the yellow arrows indicate the undegraded membranes, the white arrows indicate the osteoblasts, and the black arrows indicate the osteocytes. (HB: host bone, NB: new bone).

Histological images depicting the cranial defect area with H&E staining are illustrated in Figure 7. At the 4-week point (Figure 7A), the observation revealed the emergence of woven bone and fibrous connective tissue in the bone defect area across all groups. In the blank control group, limited new bone formation occurred at the defect area’s edge, accompanied by a sparse distribution of osteoblasts. Complete infiltration of fibrous connective tissue was evident within the defect area. Notably, the FGBM PH group exhibited a visibly thicker membrane in the defect area compared to the FGBM PHG2 group. The PLGA/nHA/Gelatin FGBM was observed within a fibrous connective tissue cyst, maintaining its original morphology and providing a conducive environment for new bone formation. The osteoblasts surrounding the new bone and osteocytes scattered in the new bone matrix were observed in the membrane-covered groups. Notably, the FGBM PHG2 group displayed the highest number of osteoblasts and the largest volume of newly-formed unmineralized bone matrix, indicative of superior osteogenic performance.

Advancing to the 8-week point (Figure 7B), remnant membranes were still discernible in the defect areas of the FGBM PH and FGBM PHG2 groups, retaining much of their original morphology despite advanced degradation. In contrast, the Bio-Gide^®^ group exhibited complete membrane degradation, with the defect area connected by fibrous connective tissue. Inflammatory cell infiltration around the FGBMs was notably less severe in the FGBM PHG2 group compared to the FGBM PH group. Relative to the 4-week assessment, a substantial increase in the volume of newly formed bone was evident in the membrane-covered groups. Conversely, the blank control group exhibited minimal change, with the defect area entirely connected by fibrous connective tissue. Within the FGBM PHG2 group, the newly formed bone appeared more mature, marked by the highest count of osteoblasts and the largest volume of newly-formed unmineralized bone matrix among the groups. Although the pace of advancement had decelerated compared to the earlier stage, it underscored a noteworthy and sustained osteogenic effect.

## 4 Discussion

### 4.1 Characterization and physicochemical properties

In this study, we successfully developed a functionally graded biomaterial membrane (FGBM) with excellent properties. Building upon the previously established bilayer structure consisting of a dense smooth phase inversion layer and a rough nanofiber layer, we incorporated a PLGA/nHA nanofiber layer into the FGBM structure to facilitate the transition between the PLGA/nHA phase inversion layer and the PLGA/nHA/Gelatin nanofiber layer, thereby promoting interlayer bonding. The incorporation of gelatin into scaffold materials can improve biocompatibility, and provide integrin-binding sites to promote cellular adhesion and proliferation (Fraioli et al., 2016). However, brittle texture, water solubility, and poor mechanical strength limit its use as a scaffold material alone (Zhang et al., 2020; Peng et al., 2021). In view of the complementary properties of polymers and gelatin, the electrospun PLGA/gelatin hybrid fibers had been widely investigated. It had been reported that electrospun fibers could be successfully prepared with PLGA/gelatin ratios of 9/1, 7/3, 5/5 and 3/7, etc. (Zheng et al., 2014; Gil-Castell et al., 2020). Nevertheless, whether the changes of PLGA/gelatin ratio have significant influence on bone tissue regeneration still lacks evidences. Based on previous studies, three different PLGA/gelatin ratios (7/3, 5/5, 3/7) were set up in this study to explore their effects on physicochemical properties and osteogenic properties of the membrane.

During synthesis, considering HFIP’s low surface tension, low boiling point, and sufficiently high dielectric constant, it was selected as an ideal solvent for electrospinning (Pérez-Nava et al., 2022). However, the presence of fluorinated alcohols can induce phase separation between PLGA and gelatin, adversely affecting fiber morphology and the electrospinning process, and accelerating polymer degradation (Shalumon et al., 2010; Feng et al., 2012). Studies have shown that acetic acid effectively addresses phase separation issues in various polymer systems. The incorporation of acetic acid modulates the conductivity and volatility of the electrospinning solution, prevents phase separation, and simplifies the electrospinning process (Feng et al., 2012; Jing et al., 2014; Gil-Castell et al., 2018). Adding a 2‰ (volume/volume) concentration of acetic acid to the PLGA/Gelatin electrospinning solution rapidly transformed the opaque solution into a transparent and uniform state. Moreover, acetic acid volatilized quickly during the electrospinning process, preserving the composition, structure, and properties of the nanofiber layer (Li et al., 2016).

Subsequent SEM and TEM observations revealed excellent surface morphology and structure of the phase inversion layer and electrospun nanofiber layer of PLGA/nHA/Gelatin FGBM. The phase inversion layer exhibited an asymmetric surface structure with one smooth and dense side and one rough and porous side. The rough surface allowed for the formation of stable bonds during subsequent electrostatic spinning on it. The nanofiber layer structure closely resembled the osteoblastic extracellular matrix (ECM), providing a favorable microenvironment for osteoblast growth. According to our observations, an increase in fiber diameter with higher gelatin content. Previous studies have reported that higher gelatin content leads to increased fiber diameter, primarily due to changes in the properties of the electrospinning solution, such as shear viscosity, surface tension, and conductivity (Howard et al., 2023). An appropriate ratio of PLGA/nHA/Gelatin electrospinning solution improved viscosity and conductivity, facilitating the formation of smooth and homogeneous nanoscale fibers.

The hydrophilic properties of biomaterial surfaces significantly influence cell adhesion, with water contact angle being a crucial indicator of surface physicochemical properties. Incorporating gelatin into the electrospun fiber layer transitions the surface from hydrophobic to hydrophilic, attributed to the microstructure of the composite fiber and the hydrophilic polar groups on the gelatin molecular chain (Ravichandran et al., 2011; Feng et al., 2012; Pozzobon et al., 2021). Although hydrophilicity improved, the water contact angle of FGBM PHG1 remained significantly higher than that of FGBM PHG2 and FGBM PHG3, indicating that most gelatin components were embedded in the continuous phase of PLGA. Increased gelatin content led to the appearance of bicontinuous phases between gelatin and PLGA, facilitating water penetration through micro-pores. Additionally, hydrogen bonding between gelatin and HFIP, along with the volatilization of HFIP during the electrospinning process, caused the migration of gelatin polar groups to the fiber surface, significantly enhancing membrane hydrophilicity, cell adhesion, and metabolic substance exchange.

Adequate tensile strength is essential for an ideal GBRM to maintain sufficient space for bone regeneration. The mechanical test results revealed a significant increase in the tensile strength of all FGBM PHG groups compared to the others, attributed to the synthesis process. To enhance interlayer bonding in FGBM, a PLGA/nHA electrospun nanofiber layer was incorporated as a transition between the phase inversion layer and the PLGA/nHA/Gelatin nanofiber layer. Due to their similar composition, the PLGA/nHA electrospun nanofiber is tightly coupled with the rough surface of the phase inversion layer (Fu et al., 2017). Furthermore, the PLGA/nHA nanofiber layer and the PLGA/nHA/Gelatin nanofiber layer share the same fiber structure, with the fibers overlapping and interweaving to form a strong bond. Additionally, we crosslinked the membrane with glutaraldehyde steam to improve the biological stability of the materials. Our study aligns with previous findings suggesting that increasing gelatin content initially enhances the tensile strength of fiber scaffolds, with a subsequent decrease. This correlation is consistent with studies such as Lee et al. (Lee et al., 2008). We suspect that this effect is mainly related to the cross-linking of fibers, where the formation of cross-linked networks between the gelatin molecular chains after cross-linking increases intermolecular forces, enhancing the tensile strength.

### 4.2 *In vitro* biodegradation properties

As a potential resorbable GBRM, it is necessary to consider the degradation performance of FGBM, as it directly impacts the barrier function and spatial maintenance capacity of the membrane. The degradation evaluation results of this study demonstrated an accelerated membrane degradation with an increase in gelatin content. With the increased proportion of gelatin, it became more exposed to the fiber surface. Despite cross-linking, a significant amount of dissolution occurred within 1 week of degradation due to the inherent hydrophilicity of gelatin. If the FGBM were implanted in the body, gelatin would degrade faster in the presence of macrophages, free radicals, and enzymes accumulate (Tracy et al., 1999). However, in this study, the FGBM remained intact for 8 weeks because of the support of the phase inversion layer. Although the electrospun fiber layer degraded rapidly, the FGBM could still provide sufficient time and space for the regeneration of bone tissue. Regarding the persistence of the *in vitro* degradation period of the phase inversion layer, this observation may be attributed to the specific ratio (75/25) of lactic and glycolic acids in PLGA, as well as the incorporation of nHA (Díaz et al., 2017). The water uptake performance and rate of the membranes with gelatin-containing increased significantly, indicating that the incorporation of gelatin would facilitate the cellular affinity of the membranes *in vivo*.

### 4.3 *In vitro* cells behaviors

The biocompatibility is significant for implantable biomaterials. In this study, osteoblast cells demonstrated enhanced adhesion and proliferation activities on gelatin-rich membranes, while the fibroblasts were effectively blocked from the outside of phase inversion layer. The gradual increase in cells and good cellular activity observed in each group throughout the culture period indicated that the FGBMs were cytocompatible. The early adhesion behavior of MC3T3-E1 cells on FGBM PHG2 and FGBM PHG3 nanofiber layers was notably more active. This was believed to be related to the higher hydrophilicity of FGBM PHG2 and FGBM PHG3, which was derived from gelatin, allowing osteoblasts to spread more easily on the surface of the nanofiber layers. Furthermore, the RGD peptide chains presented in gelatin could be recognized by integral protein receptors on cells. The polar groups on gelatin enhanced their interaction with negatively charged cells, thereby playing a role in the targeting of cells during the early adhesion and proliferation processes (Fraioli et al., 2016; Cai et al., 2020). However, compared to the other groups, the proliferation viability of cells in the FGBM PHG3 group exhibited a decline at 7 days. This decline could be attributed to the rapid degradation of gelatin, the principal component of the FGBM PHG3 membrane, and the disruption of the fibrous structure under cell culture conditions. It is essential to acknowledge potential challenges, such as the cytotoxicity of calcium phosphate particles, especially at the nanoscale to submicron scale, as the nHA particles in FGBM PHG2 were uniformly dispersed and increased fiber roughness (Sun et al., 1998; Chen et al., 2019). Further investigation into potential cytotoxic effects is therefore warranted. In contrast, the FGBM PHG2 group exhibited the highest cellular activity during the culture period, indicating that the PLGA and gelatin formed a double continuous phase interwoven structure. The hydrophobic interaction between PLGA and gelatin limited a significant amount of gelatin leaching. Even with gelatin degradation, the continuous PLGA phase was able to maintain the fiber structure stability. Furthermore, it had been demonstrated that fiber diameter influences cell adhesion, proliferation, and differentiation to some extent. The smaller the diameter of the fibers, the more cells adhered and the more spread out the cell morphology (Tian et al., 2008; Christopherson et al., 2009). The fibers with the smallest diameter were observed in FGBM PHG2, which might also contribute to their highest cell viability.

ALP is an extracellular enzyme secreted by osteoblasts and serves as a key marker of osteoblast differentiation (Li et al., 2019; Liu et al., 2021). In this study, the relevance between ALP activity and gelatin proportions was demonstrated through measurements taken at 1 and 14 days of MC3T3-E1 cell culture. Notably, the FGBM PHG2 group exhibited the highest ALP activity, whereas the FGBM PHG3 group demonstrated the lowest activity. Consistent findings were observed in ARS staining, confirming variations in cell mineral concentration between these two groups. The findings in the FGBM PHG3 group suggest that the degradation of gelatin and the lysis of nHA contributed to damage in the structure and composition of the electrospun fiber layer. This, in turn, led to a reduction in the number of cells and a decrease in the differentiation activity of individual cells. These insights underscore the importance of maintaining the stability of the fibrous structure for optimal osteogenic differentiation.

### 4.4 *In vivo* osteogenesis assessments

The *in vitro* assessments of FGBM revealed favorable mechanical properties, an appropriate biodegradation rate, and satisfactory *in vitro* biocompatibility and osteogenic ability. The FGBM PHG2 group demonstrated optimal overall *in vitro* performance, particularly in terms of osteogenic ability. Therefore, the FGBM PHG2 group was chosen as the test group to evaluate its bone regenerative ability and tissue compatibility *in vivo*. The commercial collagen membrane, Bio-Gide^®^, is a representative example and has been widely used in clinical practice (Zhang et al., 2019). Micro-CT assessments demonstrated that the FGBM PHG2 group significantly enhanced bone regeneration compared to the FGBM PH group and the commercial product (Bio-Gide^®^). Specifically, the FGBM PHG2 group had the highest bone volume of regenerated new bone (nearly 30%) to quantify the observed improvements in bone regeneration. A histological assessment of the bone defect area was performed to further investigate the mechanism by which FGBM PHG2 regenerated a significant amount of bone. Histological assessments, conducted through H&E staining, revealed that the *in vivo* degradation rate of FGBM was notably slower compared to Bio-Gide^®^. Although partial degradation was observed, FGBMs retained their original morphology even after 8 weeks of implantation, suggesting the robustness of the phase inversion layer in maintaining mechanical properties *in vivo*. The adequate tensile strength and cellular barrier function of the phase inversion layer of FGBM PHG2 prevented fibroblasts on the soft tissue side from growing into the bone defect area, and the reasonable degradation rate of FGBM created a protective environment for bone tissue regeneration. The selection of an optimal gelatin content was a critical determinant in achieving the observed positive outcomes. This nuanced choice significantly contributed to the overall success of FGBM PHG2. The relatively rapid degradation of PLGA/nHA/Gel FGBM with this specific gelatin content accelerated the release of nHA particles, thereby promoting further *in vivo* osteogenic mineralization (Wang et al., 2023). This nuanced choice in composition contributed significantly to the overall success of FGBM PHG2. Calcium ions in the degraded and released nHA particles exchanged with carboxylic acids in PLGA and gelatin, maintaining the stability of the osteogenic microenvironment and reducing the inflammatory response (Li et al., 2014). Meanwhile, the degradation-induced exposure of the intrinsic RGD peptide chain on gelatin played a pivotal role in facilitating osteogenesis. This exposure, by promoting the adhesion and proliferation of integrin-mediated osteoblast-related cells and inducing extracellular matrix (ECM) mineralization, significantly contributed to the observed positive outcomes in osteogenesis (Hsiong et al., 2009; Amjadian et al., 2016; Cai et al., 2020). Amino acids, the degradation product of gelatin, provided cellular nutrients for the local osteogenic microenvironment and facilitated osteoblast proliferation (Gu et al., 2018). The findings indicated that the FGBM PHG2 group exhibited a faster rate and greater efficacy in promoting *in vivo* osteogenesis in comparison to the FGBM PH group. This was evidenced by the FGBM PHG2 group exhibiting the largest number of osteoblasts and the largest volume of the newly-formed unmineralized bone matrix. Local inflammation in the defect area was reduced in the FGBM PHG2 group compared to the FGBM PH group. This is likely due to the incorporation of gelatin, which improves the hydrophilicity and *in vivo* biocompatibility of the FGBM. Overall, the FGBM PHG2 demonstrated excellent comprehensive performance *in vitro* and *in vivo*.

## 5 Conclusion

This study developed a novel PLGA/nHA/Gelatin functionally graded bilayer membrane through the integration of phase inversion and electrospinning methods. The investigation focused on assessing its physicochemical and biological properties and examining its impact on osteogenesis and tissue compatibility in an animal model. Results revealed that the addition of gelatin did not affect the electrospinning performance of the solution. Moreover, the resulting electrospun fiber layer tightly integrated with the phase inversion layer, displaying enhanced hydrophilic, mechanical, and biodegradation properties.

The PLGA/nHA/Gelatin FGBM with an asymmetric structure exhibited excellent tissue barrier function due to the presence of the smooth phase inversion layer. Furthermore, the nanofiber layer effectively mimicked the extracellular matrix of natural osteoblasts, which facilitated osteoblast proliferation and differentiation. Particularly, the PLGA/nHA/Gelatin FGBM with a PLGA/Gelatin mass ratio of 1:1 (FGBM PHG2) exhibited optimal osteoconductive and osteoinductive activities both *in vitro* and *in vivo*. These findings suggest its potential application in bone regeneration treatments.

## Data Availability

The raw data supporting the conclusions of this article will be made available by the authors, without undue reservation.

## References

[B1] Abe G. L. Sasaki J.-I. Katata C. Kohno T. Tsuboi R. Kitagawa H. (2020). Fabrication of novel poly(lactic acid/caprolactone) bilayer membrane for GBR application. Dent. Mater. 36 (5), 626–634. 10.1016/j.dental.2020.03.013 32224061

[B2] Aldana A. A. Abraham G. A. (2017). Current advances in electrospun gelatin-based scaffolds for tissue engineering applications. Int. J. Pharm. 523 (2), 441–453. 10.1016/j.ijpharm.2016.09.044 27640245

[B3] Amjadian S. Seyedjafari E. Zeynali B. Shabani I. (2016). The synergistic effect of nano-hydroxyapatite and dexamethasone in the fibrous delivery system of gelatin and poly(l-lactide) on the osteogenesis of mesenchymal stem cells. Int. J. Pharm. 507 (1), 1–11. 10.1016/j.ijpharm.2016.04.032 27107902

[B4] Bee S.-L. Hamid Z. A. A. (2022). Asymmetric resorbable-based dental barrier membrane for periodontal guided tissue regeneration and guided bone regeneration: a review. J. Biomed. Mater. Res. Part B Appl. Biomaterials 110 (9), 2157–2182. 10.1002/jbm.b.35060 35322931

[B5] Buser D. Urban I. Monje A. Kunrath M. F. Dahlin C. (2023). Guided bone regeneration in implant dentistry: basic principle, progress over 35 years, and recent research activities. Periodontol 2000 93 (1), 9–25. 10.1111/prd.12539 38194351

[B6] Cai S. Wu C. Yang W. Liang W. Yu H. Liu L. (2020). Recent advance in surface modification for regulating cell adhesion and behaviors. Nanotechnol. Rev. 9 (1), 971–989. 10.1515/ntrev-2020-0076

[B7] Chen F. Wang M. Wang J. Chen X. Li X. Xiao Y. (2019). Effects of hydroxyapatite surface nano/micro-structure on osteoclast formation and activity. J. Mater. Chem. B 7 (47), 7574–7587. 10.1039/C9TB01204D 31729515

[B8] Christopherson G. T. Song H. Mao H.-Q. (2009). The influence of fiber diameter of electrospun substrates on neural stem cell differentiation and proliferation. Biomaterials 30 (4), 556–564. 10.1016/j.biomaterials.2008.10.004 18977025

[B9] Dahlin C. Linde A. Gottlow J. Nyman S. (1988). Healing of bone defects by guided tissue regeneration. Plast. Reconstr. Surg. 81 (5), 672–676. 10.1097/00006534-198805000-00004 3362985

[B10] Díaz E. Puerto I. Ribeiro S. Lanceros-Mendez S. Barandiarán J. M. (2017). The influence of copolymer composition on PLGA/nHA scaffolds’ cytotoxicity and *in vitro* degradation. Nanomaterials 7 (7), 173. 10.3390/nano7070173 28684725 PMC5535239

[B11] Elgali I. Omar O. Dahlin C. Thomsen P. (2017). Guided bone regeneration: materials and biological mechanisms revisited. Eur. J. Oral Sci. 125 (5), 315–337. 10.1111/eos.12364 28833567 PMC5601292

[B12] Feng B. Tu H. Yuan H. Peng H. Zhang Y. (2012). Acetic-acid-mediated miscibility toward electrospinning homogeneous composite nanofibers of GT/PCL. Biomacromolecules 13 (12), 3917–3925. 10.1021/bm3009389 23131188

[B13] Fraioli R. Dashnyam K. Kim J.-H. Perez R. A. Kim H.-W. Gil J. (2016). Surface guidance of stem cell behavior: chemically tailored co-presentation of integrin-binding peptides stimulates osteogenic differentiation *in vitro* and bone formation *in vivo* . Acta Biomater. 43, 269–281. 10.1016/j.actbio.2016.07.049 27481289

[B14] Fu L. Wang Z. Dong S. Cai Y. Ni Y. Zhang T. (2017). Bilayer poly(lactic-co-glycolic acid)/nano-hydroxyapatite membrane with barrier function and osteogenesis promotion for guided bone regeneration. Mater. (Basel) 10 (3), 257. 10.3390/ma10030257 PMC5503363 28772618

[B15] Gautam S. Sharma C. Purohit S. D. Singh H. Dinda A. K. Potdar P. D. (2021). Gelatin-polycaprolactone-nanohydroxyapatite electrospun nanocomposite scaffold for bone tissue engineering. Mater. Sci. Eng. C 119, 111588. 10.1016/j.msec.2020.111588 33321633

[B16] Gil-Castell O. Badia J. D. Ontoria-Oviedo I. Castellano D. Sepúlveda P. Ribes-Greus A. (2020). Polycaprolactone/gelatin-based scaffolds with tailored performance: *in vitro* and *in vivo* validation. Mater. Sci. Eng. C 107, 110296. 10.1016/j.msec.2019.110296 31761169

[B17] Gil-Castell O. Badia J. D. Ribes-Greus A. (2018). Tailored electrospun nanofibrous polycaprolactone/gelatin scaffolds into an acid hydrolytic solvent system. Eur. Polym. J. 101, 273–281. 10.1016/j.eurpolymj.2018.02.030

[B18] Gu Y. Bai Y. Zhang D. (2018). Osteogenic stimulation of human dental pulp stem cells with a novel gelatin-hydroxyapatite-tricalcium phosphate scaffold. J. Biomed. Mater. Res. Part A 106 (7), 1851–1861. 10.1002/jbm.a.36388 29520937

[B19] Howard C. J. Paul A. Duruanyanwu J. Sackho K. Campagnolo P. Stolojan V. (2023). The manufacturing conditions for the direct and reproducible formation of electrospun PCL/gelatine 3D structures for tissue regeneration. Nanomaterials 13 (24), 3107. 10.3390/nano13243107 38133004 PMC10745430

[B20] Hsiong S. X. Boontheekul T. Huebsch N. Mooney D. J. (2009). Cyclic arginine-glycine-aspartate peptides enhance three-dimensional stem cell osteogenic differentiation. Tissue Eng. Part A 15 (2), 263–272. 10.1089/ten.tea.2007.0411 18783323 PMC2774232

[B21] Ji W. Yang F. Seyednejad H. Chen Z. Hennink W. E. Anderson J. M. (2012). Biocompatibility and degradation characteristics of PLGA-based electrospun nanofibrous scaffolds with nanoapatite incorporation. Biomaterials 33 (28), 6604–6614. 10.1016/j.biomaterials.2012.06.018 22770568

[B22] Jin S. Sun F. Zou Q. Huang J. Zuo Y. Li Y. (2019). Fish collagen and hydroxyapatite reinforced poly(lactide-co-glycolide) fibrous membrane for guided bone regeneration. Biomacromolecules 20 (5), 2058–2067. 10.1021/acs.biomac.9b00267 31009574

[B23] Jin S. Xia X. Huang J. Yuan C. Zuo Y. Li Y. (2021). Recent advances in PLGA-based biomaterials for bone tissue regeneration. Acta Biomater. 127, 56–79. 10.1016/j.actbio.2021.03.067 33831569

[B24] Jing X. Salick M. R. Cordie T. Mi H.-Y. Peng X.-F. Turng L.-S. (2014). Electrospinning homogeneous nanofibrous poly(propylene carbonate)/gelatin composite scaffolds for tissue engineering. Industrial and Eng. Chem. Res. 53 (22), 9391–9400. 10.1021/ie500762z

[B25] Lee J. Tae G. Kim Y. H. Park I. S. Kim S.-H. Kim S. H. (2008). The effect of gelatin incorporation into electrospun poly(l-lactide-co-ɛ-caprolactone) fibers on mechanical properties and cytocompatibility. Biomaterials 29 (12), 1872–1879. 10.1016/j.biomaterials.2007.12.029 18234330

[B26] Li D. Chen W. Sun B. Li H. Wu T. Ke Q. (2016). A comparison of nanoscale and multiscale PCL/gelatin scaffolds prepared by disc-electrospinning. Colloids Surf. B Biointerfaces 146, 632–641. 10.1016/j.colsurfb.2016.07.009 27429297

[B27] Li D. Sun H. Jiang L. Zhang K. Liu W. Zhu Y. (2014). Enhanced biocompatibility of PLGA nanofibers with gelatin/nano-hydroxyapatite bone biomimetics incorporation. ACS Appl. Mater. and Interfaces 6 (12), 9402–9410. 10.1021/am5017792 24877641

[B28] Li N. Zhou L. Xie W. Zeng D. Cai D. Wang H. (2019). Alkaline phosphatase enzyme-induced biomineralization of chitosan scaffolds with enhanced osteogenesis for bone tissue engineering. Chem. Eng. J. 371, 618–630. 10.1016/j.cej.2019.04.017

[B29] Li P. Li Y. Kwok T. Yang T. Liu C. Li W. (2021). A bi-layered membrane with micro-nano bioactive glass for guided bone regeneration. Colloids Surfaces B Biointerfaces 205, 111886. 10.1016/j.colsurfb.2021.111886 34091371

[B30] Lian M. Han Y. Sun B. Xu L. Wang X. Ni B. (2020). A multifunctional electrowritten bi-layered scaffold for guided bone regeneration. Acta Biomater. 118, 83–99. 10.1016/j.actbio.2020.08.017 32853801

[B31] Lian M. Sun B. Qiao Z. Zhao K. Zhou X. Zhang Q. (2019). Bi-layered electrospun nanofibrous membrane with osteogenic and antibacterial properties for guided bone regeneration. Colloids Surfaces B Biointerfaces 176, 219–229. 10.1016/j.colsurfb.2018.12.071 30623809

[B32] Lin X. Patil S. Gao Y.-G. Qian A. (2020). The bone extracellular matrix in bone formation and regeneration. Front. Pharmacol. 11, 757. 10.3389/fphar.2020.00757 32528290 PMC7264100

[B33] Liu W. Dong X. Qin H. Sui L. Wang J. (2021). Three-dimensional porous reduced graphene oxide/hydroxyapatite membrane for guided bone regeneration. Colloids Surfaces B Biointerfaces 208, 112102. 10.1016/j.colsurfb.2021.112102 34509086

[B34] Liu Y. Li W. (2019). Nature-Inspired multifunctional bilayer architecture advances bone defect repair. Chem 5 (10), 2515–2517. 10.1016/j.chempr.2019.09.004

[B35] Mizraji G. Davidzohn A. Gursoy M. Gursoy U. K. Shapira L. Wilensky A. (2023). Membrane barriers for guided bone regeneration: an overview of available biomaterials. Periodontol 2000 93 (1), 56–76. 10.1111/prd.12502 37855164

[B36] Naenni N. Stucki L. Hüsler J. Schneider D. Hämmerle C. H. F. Jung R. E. (2021). Implants sites with concomitant bone regeneration using a resorbable or non-resorbable membrane result in stable marginal bone levels and similar profilometric outcomes over 5 years. Clin. Oral Implants Res. 32 (8), 893–904. 10.1111/clr.13764 33977571

[B37] Nahid R. Bansal M. Pandey S. (2022). Horizontal bone augmentation using two membranes at dehisced implant sites: a randomized clinical study. J. Oral Biol. Craniofacial Res. 12 (5), 487–491. 10.1016/j.jobcr.2022.06.003 PMC9207285 35733847

[B38] Naik A. Shepherd D. V. Shepherd J. H. Best S. M. Cameron R. E. (2017). The effect of the type of HA on the degradation of PLGA/HA composites. Mater. Sci. Eng. C 70, 824–831. 10.1016/j.msec.2016.09.048 27770960

[B39] Omar O. Elgali I. Dahlin C. Thomsen P. (2019). Barrier membranes: more than the barrier effect? J. Clin. Periodontology 46 (S21), 103–123. 10.1111/jcpe.13068 PMC6704362 30667525

[B40] Patil S. Bhandi S. Bakri M. M. H. Albar D. H. Alzahrani K. J. Al-Ghamdi M. S. (2023). Evaluation of efficacy of non-resorbable membranes compared to resorbable membranes in patients undergoing guided bone regeneration. Heliyon 9 (3), e13488. 10.1016/j.heliyon.2023.e13488 36942236 PMC10024103

[B41] Peng T. Zhu J. Huang T. Jiang C. Zhao F. Ge S. (2021). Facile preparation for gelatin/hydroxyethyl 2 composite aerogel with good mechanical strength, heat insulation, and water resistance. J. Appl. Polym. Sci. 138 (23), 50539. 10.1002/app.50539

[B42] Pérez-Nava A. Reyes-Mercado E. González-Campos J. B. (2022). Production of chitosan nanofibers using the HFIP/acetic acid mixture as electrospinning solvent. Chem. Eng. Process. - Process Intensif. 173, 108849. 10.1016/j.cep.2022.108849

[B43] Pozzobon L. G. Sperling L. E. Teixeira C. E. Malysz T. Pranke P. (2021). Development of a conduit of PLGA-gelatin aligned nanofibers produced by electrospinning for peripheral nerve regeneration. Chemico-Biological Interact. 348, 109621. 10.1016/j.cbi.2021.109621 34450165

[B44] Qin X. Pan H. Yang K. Xie W. Yang G. Wang J. (2023). Biodegradable and biocompatible alginate/gelatin/MXene composite membrane with efficient osteogenic activity and its application in guided bone regeneration. J. Biomaterials Sci. 34, 1843–1857. 10.1080/09205063.2023.2187987 36869856

[B45] Ravichandran R. Ng C. C. H. Liao S. Pliszka D. Raghunath M. Ramakrishna S. (2011). Biomimetic surface modification of titanium surfaces for early cell capture by advanced electrospinning. Biomed. Mater. 7 (1), 015001. 10.1088/1748-6041/7/1/015001 22156014

[B46] Retzepi M. Donos N. (2010). Guided Bone Regeneration: biological principle and therapeutic applications. Clin. Oral Implants Res. 21 (6), 567–576. 10.1111/j.1600-0501.2010.01922.x 20666785

[B47] Rider P. Kačarević Ž. P. Elad A. Tadic D. Rothamel D. Sauer G. (2022). Biodegradable magnesium barrier membrane used for guided bone regeneration in dental surgery. Bioact. Mater. 14, 152–168. 10.1016/j.bioactmat.2021.11.018 35310351 PMC8892166

[B48] Salhotra A. Shah H. N. Levi B. Longaker M. T. (2020). Mechanisms of bone development and repair. Nat. Rev. Mol. Cell Biol. 21 (11), 696–711. 10.1038/s41580-020-00279-w 32901139 PMC7699981

[B49] Shah A. T. Zahid S. Ikram F. Maqbool M. Chaudhry A. A. Rahim M. I. (2019). Tri-layered functionally graded membrane for potential application in periodontal regeneration. Mater. Sci. Eng. C 103, 109812. 10.1016/j.msec.2019.109812 31349482

[B50] Shalumon K. T. Anulekha K. H. Girish C. M. Prasanth R. Nair S. V. Jayakumar R. (2010). Single step electrospinning of chitosan/poly(caprolactone) nanofibers using formic acid/acetone solvent mixture. Carbohydr. Polym. 80 (2), 413–419. 10.1016/j.carbpol.2009.11.039

[B51] Sheikh Z. Qureshi J. Alshahrani A. M. Nassar H. Ikeda Y. Glogauer M. (2017). Collagen based barrier membranes for periodontal guided bone regeneration applications. Odontology 105 (1), 1–12. 10.1007/s10266-016-0267-0 27613193

[B52] Shi J.-Y. Montero E. Wu X.-Y. Palombo D. Wei S.-M. Sanz-Sánchez I. (2022). Bone preservation or augmentation simultaneous with or prior to dental implant placement: a systematic review of outcomes and outcome measures used in clinical trials in the last 10 years. Clin. Oral Implants Res. 34 (S25), 68–83. 10.1111/clr.13953 35817421

[B53] Sun J.-S. Liu H.-C. Hong-Shong Chang W. Li J. Lin F.-H. Tai H.-C. (1998). Influence of hydroxyapatite particle size on bone cell activities: an *in vitro* study. J. Biomed. Mater. Res. 39 (3), 390–397. 10.1002/(SICI)1097-4636(19980305)39:3<390::AID-JBM7>3.0.CO;2-E 9468047

[B54] Testori T. Weinstein T. Scutellà F. Wang H. L. Zucchelli G. (2018). Implant placement in the esthetic area: criteria for positioning single and multiple implants. Periodontol 2000 77 (1), 176–196. 10.1111/prd.12211 29484714

[B55] Tian F. Hosseinkhani H. Hosseinkhani M. Khademhosseini A. Yokoyama Y. Estrada G. G. (2008). Quantitative analysis of cell adhesion on aligned micro- and nanofibers. J. Biomed. Mater. Res. Part A 84A (2), 291–299. 10.1002/jbm.a.31304 17607759

[B56] Tracy M. A. Ward K. L. Firouzabadian L. Wang Y. Dong N. Qian R. (1999). Factors affecting the degradation rate of poly(lactide-co-glycolide) microspheres *in vivo* and *in vitro* . Biomaterials 20 (11), 1057–1062. 10.1016/S0142-9612(99)00002-2 10378806

[B57] Wang H. Li X. Lai S. Cao Q. Liu Y. Li J. (2023). Construction of vascularized tissue engineered bone with nHA-coated BCP bioceramics loaded with peripheral blood-derived MSC and EPC to repair large segmental femoral bone defect. ACS Appl. Mater. and Interfaces 15 (1), 249–264. 10.1021/acsami.2c15000 36548196

[B58] Xiang J. Li Y. Ren M. He P. Liu F. Jing Z. (2022). Sandwich-like nanocomposite electrospun silk fibroin membrane to promote osteogenesis and antibacterial activities. Appl. Mater. Today 26, 101273. 10.1016/j.apmt.2021.101273

[B59] Xu C. Liu Z. Chen X. Gao Y. Wang W. Zhuang X. (2024). Bone tissue engineering scaffold materials: fundamentals, advances, and challenges. Chin. Chem. Lett. 35 (2), 109197. 10.1016/j.cclet.2023.109197

[B60] Yoshimoto I. Sasaki J.-I. Tsuboi R. Yamaguchi S. Kitagawa H. Imazato S. (2018). Development of layered PLGA membranes for periodontal tissue regeneration. Dent. Mater. 34 (3), 538–550. 10.1016/j.dental.2017.12.011 29310906

[B61] Zhang K.-R. Gao H.-L. Pan X.-F. Zhou P. Xing X. Xu R. (2019). Multifunctional bilayer nanocomposite guided bone regeneration membrane. Matter 1 (3), 770–781. 10.1016/j.matt.2019.05.021

[B62] Zhang L. Dong Y. Zhang N. Shi J. Zhang X. Qi C. (2020). Potentials of sandwich-like chitosan/polycaprolactone/gelatin scaffolds for guided tissue regeneration membrane. Mater. Sci. Eng. C 109, 110618. 10.1016/j.msec.2019.110618 32228889

[B63] Zheng M. Wang X. Yue O. Hou M. Zhang H. Beyer S. (2021). Skin-inspired gelatin-based flexible bio-electronic hydrogel for wound healing promotion and motion sensing. Biomaterials 276, 121026. 10.1016/j.biomaterials.2021.121026 34298443

[B64] Zheng R. Duan H. Xue J. Liu Y. Feng B. Zhao S. (2014). The influence of Gelatin/PCL ratio and 3-D construct shape of electrospun membranes on cartilage regeneration. Biomaterials 35 (1), 152–164. 10.1016/j.biomaterials.2013.09.082 24135269

