# Supplementary material for: A novel functionally graded bilayer membrane with excellent barrier function and in vivo osteogenesis promotion for guided bone regeneration
Source: Front Pharmacol. 2024 Sep 27;15:1453036. doi: 10.3389/fphar.2024.1453036 (PMC11472855; doi:10.3389/fphar.2024.1453036)
Supplement: Supplementary file 1 [file Image1.pdf]

Supplementary Material

A Novel Functionally Graded Bilayer Membrane with Excellent Barrier Function and In Vivo Osteogenesis Promotion for Guided Bone Regeneration

Junxuan Li, Jiaxin Ding, Tao Zhou, Bolun Li, Jingjing Wang, Hanchi Wang *, and Li Fu*

Department of Oral Implantology, Jilin Provincial Key Laboratory of Tooth Development and Bone Remodeling, Hospital of Stomatology, Jilin University, Changchun 130021, China.

*** Correspondence:**

Corresponding Authors

Hanchi Wang

Department of Oral Implantology, Jilin Provincial Key Laboratory of Tooth Development and Bone Remodeling, Hospital of Stomatology, Jilin University, 1500 Qinghua Road, Chaoyang District, Changchun, 130021, China

Email: whc23@jlu.edu.cn

Li Fu

Department of Oral Implantology, Jilin Provincial Key Laboratory of Tooth Development and Bone Remodeling, Hospital of Stomatology, Jilin University, 1500 Qinghua Road, Chaoyang District, Changchun, 130021, China

Email: fuli1127@jlu.edu.cn

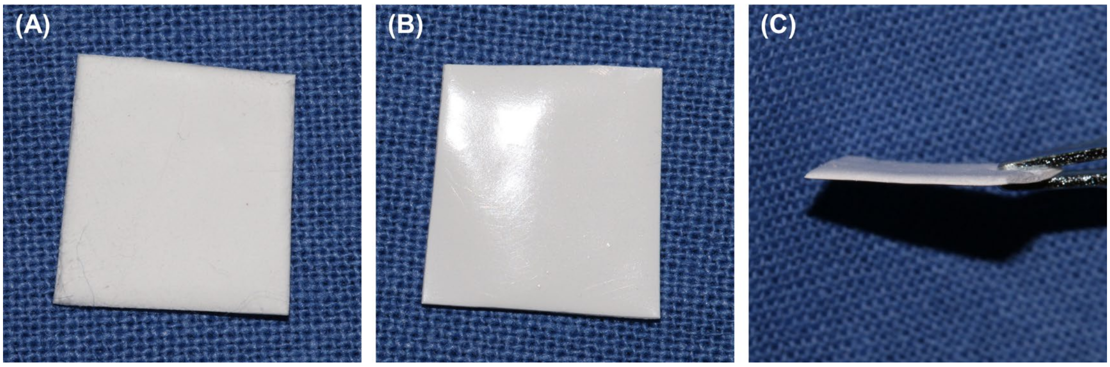

FIGURE S1. Representative images of the macroscopic morphology of the fabricated FGBM. (A) The rough surface of the electrospun fiber layer belonging to FGBM. (B) The smooth surface of the phase inversion layer belonging to FGBM. (C) The cross-sectional morphology of FGBM.

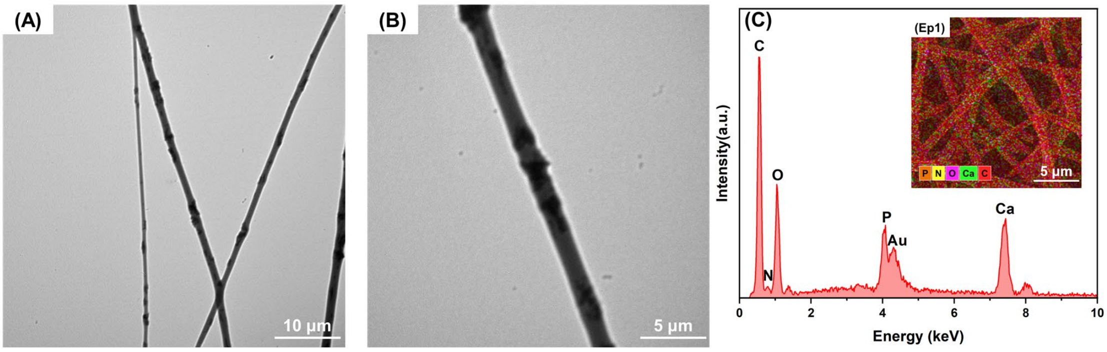

FIGURE S2. Representative TEM images and energy-dispersive X-ray (EDX) spectrum and elemental mapping image of the PLGA/nHA/Gelatin nanofibers. (A) TEM image of the nanofibers, scale bar = 10 μm. (B) TEM image of the nanofiber, scale bar = 5 μm. (C) EDX spectrum image of the PLGA/nHA/Gelatin nanofiber layer, scale bar =5 μm. Ep1 is an elemental mapping image of the nanofiber layer.

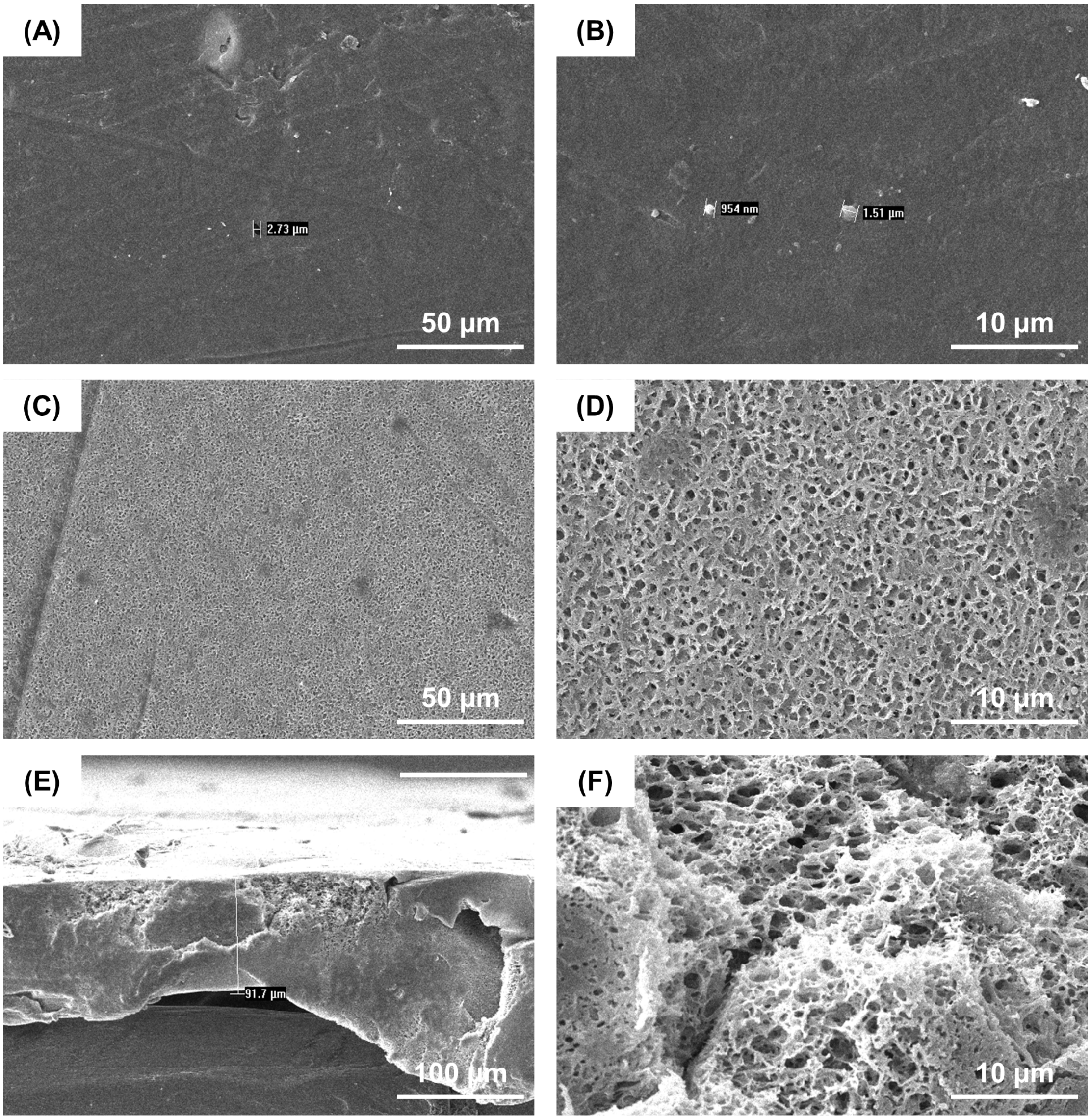

FIGURE S3. Representative SEM images of surface morphology and structure of individual phase inversion membrane (PIM + 5%nHA). **(A&B)** The smooth surface of PIM. **(C&D)** The rough surface with the average pore diameter being about 2-3 µm. **(E&F)** The cross-sectional thickness of the PIM was about 90-100 µm with porous structure.

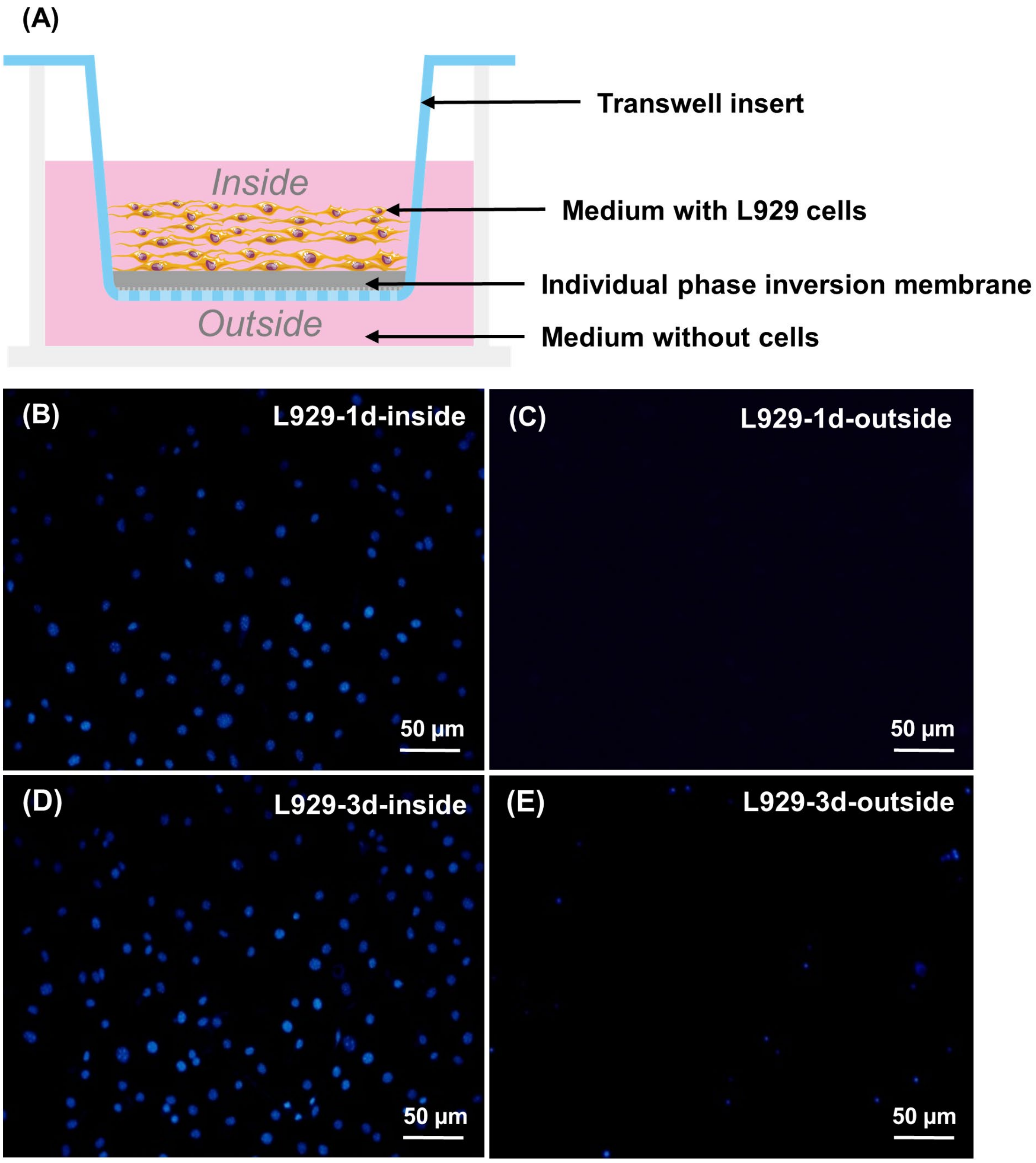

FIGURE S4. Schematic representation of in vitro barrier function assay and representative fluorescence images of L929 cells cultured on the both sides of PIM. **(A)** Schematic diagram of a barrier function testing device. **(B&D)** An increased number of cells were observed in the medium on the surface side of the PIM (inside) at 1 and 3 days of culture. **(C&E)** Few cells were observed in the medium under the PIM (outside) at 1 and 3 days of culture.

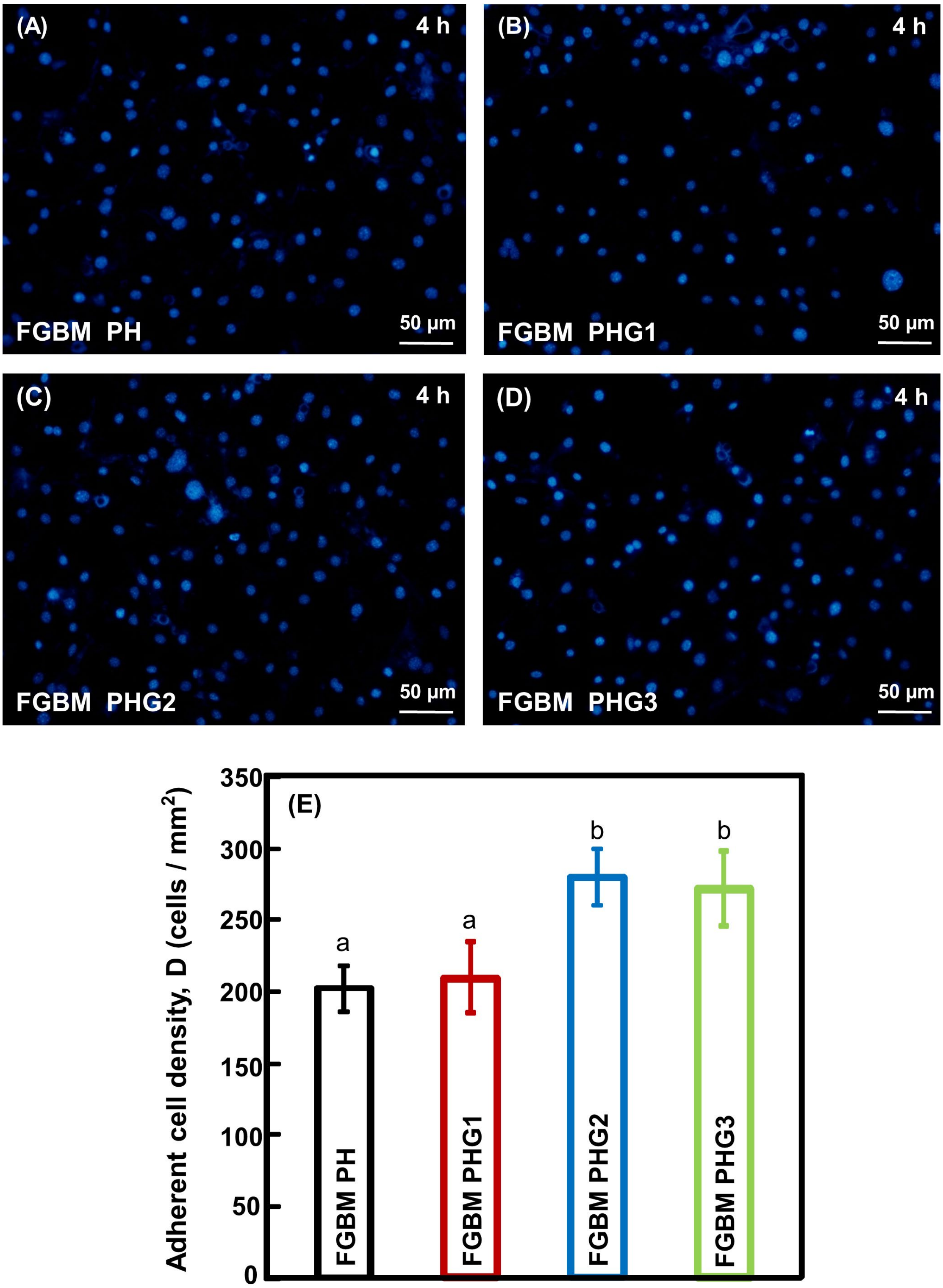

FIGURE S5. Representative fluorescent images of MC3T3-E1 cells adhesion onto the electrospun fiber layer of FGBMs at 4 h of culture. **(A-D)** Representative fluorescent images of **(A)** FGBM PH; **(B)** FGBM PHG1; **(C)** FGBM PHG2 and **(D)** FGBM PHG3. **(E)** cell density on the electrospun fiber layer of the four groups of FGBM. Different letters above the bar graphs represent statistical differences among groups ($P < 0.05$).
